# Accelerating Antimicrobial Peptide Discovery for WHO Priority Pathogens through Predictive and Interpretable Machine Learning Models

**DOI:** 10.1021/acsomega.3c08676

**Published:** 2024-02-13

**Authors:** Cheng-Ting Tsai, Chia-Wei Lin, Gen-Lin Ye, Shao-Chi Wu, Philip Yao, Ching-Ting Lin, Lei Wan, Hui-Hsu Gavin Tsai

**Affiliations:** † Department of Chemistry, National Central University, No. 300, Zhongda Road, Zhongli District, Taoyuan 32001, Taiwan; ‡ Research Center of New Generation Light Driven Photovoltaic Modules, National Central University, Taoyuan 32001, Taiwan; § School of Chinese Medicine, China Medical University, No. 91 Hsueh-Shih Road, Taichung 40402, Taiwan; ∥ Aurora High School, 109 W Pioneer Trail, Aurora, Ohio 44202, United States

## Abstract

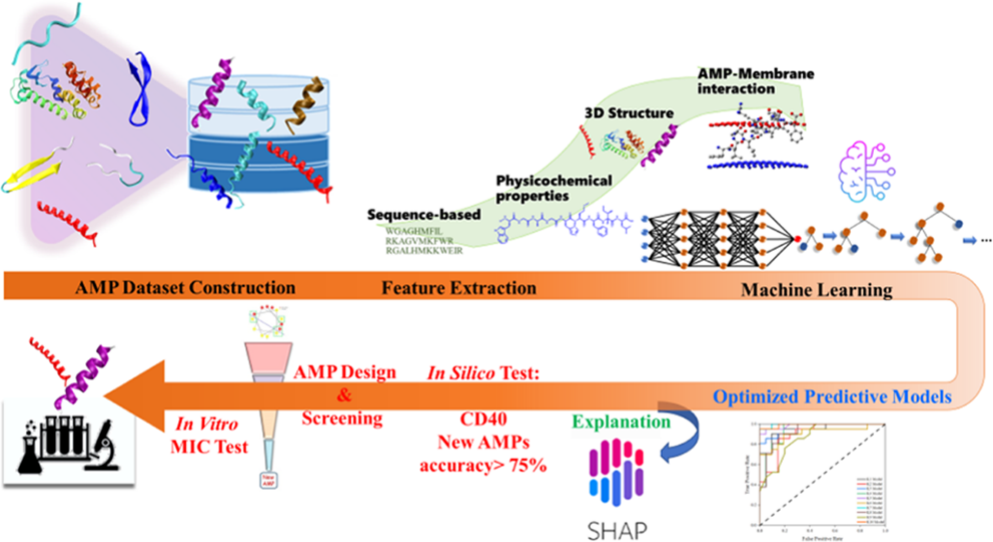

The escalating menace of multidrug-resistant (MDR) pathogens necessitates a paradigm shift from conventional antibiotics to innovative alternatives. Antimicrobial peptides (AMPs) emerge as a compelling contender in this arena. Employing *in silico* methodologies, we can usher in a new era of AMP discovery, streamlining the identification process from vast candidate sequences, thereby optimizing laboratory screening expenditures. Here, we unveil cutting-edge machine learning (ML) models that are both predictive and interpretable, tailored for the identification of potent AMPs targeting World Health Organization’s (WHO) high-priority pathogens. Furthermore, we have developed ML models that consider the hemolysis of human erythrocytes, emphasizing their therapeutic potential. Anchored in the nuanced physical–chemical attributes gleaned from the three-dimensional (3D) helical conformations of AMPs, our optimized models have demonstrated commendable performance—boasting an accuracy exceeding 75% when evaluated against both low-sequence-identified peptides and recently unveiled AMPs. As a testament to their efficacy, we deployed these models to prioritize peptide sequences stemming from PEM-2 and subsequently probed the bioactivity of our algorithm-predicted peptides vis-à-vis WHO’s priority pathogens. Intriguingly, several of these new AMPs outperformed the native PEM-2 in their antimicrobial prowess, thereby underscoring the robustness of our modeling approach. To elucidate ML model outcomes, we probe via Shapley Additive exPlanations (SHAP) values, uncovering intricate mechanisms guiding diverse actions against bacteria. Our state-of-the-art predictive models expedite the design of new AMPs, offering a robust countermeasure to antibiotic resistance. Our prediction tool is available to the public at https://ai-meta.chem.ncu.edu.tw/amp-meta.

## Introduction

The increased global prevalence of multidrug-resistant (MDR) pathogens is a significant concern in the field of global public healthcare.^1^ The alarming escalation of MDR can be attributed to their pervasive use, coupled with the prevalent gene transfer within the food supply chain,^2^ the presence of commensal bacteria in community settings in low- and middle-income countries,^3^ and the intricate, region-specific factors in developing countries.^4^ Unfortunately, the discovery of novel antibiotics has been on a steady decline, necessitating the urgent development of new therapeutic strategies to effectively control infections. Pathogenic bacteria possess efflux pumps and porin, which represent viable targets for the discovery of new drugs. However, these pathogens have evolved extensive antibiotic resistance mechanisms, rendering most conventional antibiotic pathways ineffective. An alternative and promising approach involves targeting and disrupting the bacterial membrane, as this strategy has the potential to circumvent many of these antibiotic resistance pathways. Antimicrobial peptides (AMPs) provide an alternative to traditional antibiotics by targeting the bacterial membrane to control infections.^5^ AMPs exhibit a broad spectrum of antimicrobial activity at low micromolar concentrations. These membrane-active peptides possess specific characteristics such as amphipathicity, positive charge (with an average net charge of 3.32),^6^ an abundance of bulky hydrophobic and aromatic amino acids (such as Phe, Tyr, and Trp), as well as residues of Cys, Gly, and Lys.^7^ The amino acid sequences of AMPs give rise to a wide range of physicochemical properties, resulting in diverse structural variations.^6,7^ Over the past century, more than 1000 AMPs have been discovered.^8−10^

AMPs possess the ability to interact with the bacterial membrane, resulting in the disruption of the cellular bilayer boundary. Several models, including the carpet model, pore formation models, and in-plane diffusion or partial insertion model, have been proposed to elucidate the mechanisms by which AMPs exert their disruptive effects on lipid membranes.^11,12^ However, despite extensive research, the molecular mechanisms underlying the antimicrobial activity and cytotoxicity of AMPs remain poorly understood. This lack of understanding is primarily attributed to the fact that AMPs are not naturally designed to interact with specific targets. Consequently, a comprehensive characterization of common activity and cytotoxicity motifs associated with AMPs has yet to be established. The absence of well-defined structure–function relationships, encompassing both activity and cytotoxicity, hampers the fundamental comprehension of the molecular mechanisms governing antimicrobial activity. A major obstacle in elucidating the underlying action mechanisms of AMPs lies in the dynamic nature of structure–function relationships^,^^13−15^ particularly those targeting fluid membranes.

A rational approach to enhance the effectiveness of peptide antibiotic analogues can be pursued by considering various factors such as amphipathicity, charge, helicity, hydrophobicity, and lipophilicity.^16,17^ Lipidation of AMPs using 10–12 carbon chains chemically increases hydrophobicity, promotes membrane insertion and pore formation, resulting in bacterial membrane destabilization and fluid leakage.^17^ Additionally, the incorporation of repeating motifs such as arginine–tryptophan^18^ and tryptophan–leucine–lysine repeats^19^ has shown promise in peptide design. Certain of these AMP design principles rely solely on sequences, while others hinge on structure–function relationships.^13−15^ Nevertheless, the effectiveness of structure-based design is curtailed by the dynamic nature of AMP action mechanisms on bacterial membranes and a restricted understanding of sequence-induced structural changes.

Machine learning (ML) algorithms offer an alternative approach to designing AMPs and have proven effective by leveraging diverse sequence-level, residue-level, structural, or physicochemical features in conjunction with extensive AMP databases.^9,10,20,21^ These approaches have successfully enabled the screening and correlation through sequences of AMPs with their antimicrobial activity.^22−26^ Moreover, several AMP prediction web servers such as CAMP_R4_,^26^ iFeature,^27^ iAMP-2L,^28^ iAMPpred,^29^ and AMPlify^30^ have been made available to the public, enabling researchers to assess the activity of their designed peptides. For example, the CAMP_R4_ Web site, developed by Idicula-Thomas and colleagues,^26^ provides four distinct classifiers for AMPs: random forest (RF), support vector machine (SVM), discriminant analysis (DA), and single layer forward neural network (ANN). These classifiers utilize features derived from amino acid composition (AAC), physicochemical, and structural properties. ML can be scaled to efficiently screen millions of sequences or more *in silico*. The efficacies of AMPs developed through ML-based approaches are comparable to those obtained through structure-guided design and other computational methods,^15,25,31^ underscoring that the full potential of ML approaches in this context has yet to be fully realized.

Numerous factors contribute to the limited progress of current ML methods in advancing AMP design. First, many ML models have been primarily constructed to discriminate between AMPs and non-AMPs, neglecting the consideration of bioactivity. Although these strategies are valuable for genome sequence screening,^32,33^ they are inadequate for identifying potent AMPs. Additionally, specific bacterial strains targeted by the identified AMPs remain ambiguous, impeding subsequent drug development efforts. This issue can be alleviated by tailoring ML models to target specific bacterial strains and by constraining the positive training set to encompass low minimum inhibitory concentration (MIC) values.^22^ Second, the evaluation of most ML models’ performance relies on test sets derived from the original data set, potentially introducing sequence identities akin to the training set. The utilization of an independent *in silico* test set is imperative to address this limitation prior to *in vitro* testing. Third, the majority of ML models have been founded solely on sequence information, thereby confining their understanding to sequence–function relationships. Incorporating the physicochemical properties based on three-dimensional structures of peptides into the models could facilitate their training to capture structure–function relationships, thus enabling a more accurate exploration of the expansive landscape of AMP activity.^31,34^ Fourth, the prominence of AMP design investigations has primarily revolved around antimicrobial efficacy, overshadowing the relatively diminished focus on hemolytic potential. An ML model capable of accurately predicting hemolysis tendencies in peptides stands to expedite the AMP discovery process. Notably, recent work by Reymond et al. has harnessed recurrent neural networks (RNNs) for the development of concise, nonhemolytic AMPs targeting pathogens such as *Pseudomonas aeruginosa*, *Acinetobacter baumannii*, and methicillin-resistant *Staphylococcus aureus* (MRSA).^35^ Fifth, the complexity and opacity of ML models, often referred to as “black boxes,” pose challenges in comprehending their prediction rationale. Hence, the integration of explainable ML models can provide valuable insights for formulating AMP design guidelines in future endeavors.

In this study, we established an ML workflow designed to tackle the aforementioned issues. We have developed predictive models for AMPs using ML algorithms specifically tailored for three bacterial strains: *Escherichia coli* ATCC 25922, *P. aeruginosa* ATCC 27853, and *S. aureus* ATCC 25923. Notably, *P. aeruginosa* and *S. aureus* are recognized as WHO priority pathogens. Additionally, we have created a predictive model for the hemolysis of human erythrocytes for potential therapeutic applications. To identify potent AMPs for drug development, we considered peptides with low minimum inhibitory concentration (MIC) values (<10 μg/mL) as the positive training set. To capture specific peptide interactions, we incorporated 3D helical structures obtained through Alphafold 2^36^ into our ML algorithms. Furthermore, we utilized a membrane model that considers essential characteristics of bacterial membranes, enabling us to analyze peptide–membrane interactions. The results of the optimized predictive models were interpreted in terms of Shapley Additive exPlanations (SHAP)^37^ values, providing insights into the underlying action mechanisms against different bacteria. To validate the performance of our models, we tested them using a data set with low-sequence dependence (<40%) and evaluated their efficacy against recently published AMPs. Additionally, based on the predictions of our models, we engineered seven peptides using PEM-2 as the basis, with targeting against *E. coli*, *P. aeruginosa* ATCC 27853, and *PA01*. These engineered peptides were subjected to *in vitro* MIC testing, and some of them demonstrated improved antimicrobial activity, thereby validating the effectiveness of our predictive models. The application of these models has the potential to expedite the discovery and development of effective AMPs to combat bacterial infections.

## Methods

### Constructing a Training Database: Strategies and Considerations

This study aimed to develop prediction tools for designing AMPs specific to *E. coli*, *P. aeruginosa*, and *S. aureus*. The selection of these bacteria was based on the WHO’s list of bacteria categorized as critically or highly prioritized for the development of new antibiotics.^38^ Among the bacteria listed, *P. aeruginosa* was classified as a Priority 1 (CRITICAL) bacterium, while *S. aureus* fell under Priority 2 (HIGH). The AMP data utilized in this research were collected from three databases: the Database of Antimicrobial Activity and Structure of Peptides (DBAASP),^10^ the Antimicrobial Peptide Database (APD3),^9^ and the Data Repository of Antimicrobial Peptides (DRAMP).^21^ The AMP data obtained from the DBAASP database covered the period until Sept 13, 2022, while the data from the APD3 database encompassed the time frame until June 13, 2022. The AMP data collected from the DRAMP database spanned until July 4, 2022.

To create strain-specific data sets, we assembled three data sets, namely, CD90_E_25922, CD90_P_27853, and CD90_S_25923, based on the abundant occurrence of AMPs in the aforementioned databases. Additionally, we constructed a data set named CD90_HE, which focused on AMPs active against human erythrocytes. CD90_E_25922 contained AMPs that exhibited activity against *E. coli* ATCC 25922, with redundancy eliminated based on sequence identity being above 90%. CD90_P_27853 encompassed AMPs effective against *P. aeruginosa* ATCC 27853, with redundancy removed using a sequence identity threshold of 90%. Similarly, CD90_S_25923 consisted of AMPs active against *S. aureus* ATCC 25923, and redundancy was eliminated based on a sequence identity larger than 90%. Finally, CD90_HE included AMPs targeting human erythrocytes, with redundancy filtered out using a sequence identity larger than 90%.

According to APD3,^9^ which provides statistical information on the 3D structures of AMPs, the most prevalent structural class is the helical structure. Helical structures facilitate the calculation of meaningful 3D structure-based features such as hydrophobic sub-moments and yield consistent features derived from sequences, such as intrapeptide pair-wise interactions. Consequently, physicochemical-based prediction models for the helical class of AMPs are expected to possess greater statistical reliability compared to models developed for other 3D structural classes, disordered structures, or mixed 3D structure data sets. Considering this, we devised new physicochemical-based predictive models for helical AMPs that exhibit activity against two Gram-negative bacterial strains (*E. coli* ATCC 25922 and *P. aeruginosa* ATCC 27853), one Gram-positive bacterial strain (*S. aureus* ATCC 25923), as well as human red blood cell hemolysis. We relied on the impressive capabilities of Alphafold 2 (AF2),^36^ a highly potent protein structure prediction tool. AF2 has significantly enhanced the accuracy of predicting protein 3D structures directly from amino acid sequences, achieving precision at the atomic level.^36,39,40^ The helicity of AMP structures predicted by AF2^36,39^ was examined by the Definition of Secondary Structure of Proteins (DSSP) algorithm.^41^ We consider AMPs with helical structures greater than 60% as helical structures. Furthermore, in view of the synthesis time and cost considerations, we limited the peptide length to be between 5 and 30 amino acids, excluding AMPs containing D-form amino acids from our data set. Additionally, anionic AMPs with a net negative charge smaller than zero were excluded from our data set.

To identify potent AMPs, we established specific conditions for the positive data set. In this case, the criterion for inclusion was an MIC of antibacterial activity that was less than or equal to 10 μg/mL. We deliberately chose a lower MIC value compared to previous studies (25^20^ and 32 μg/mL^22,35^) to enhance the prediction of AMPs with low MIC values, which are particularly valuable for potent applications. Diverging from conventional approaches where the negative set comprises non-AMPs, our negative set consisted of AMPs with high MIC values (>100 μg/mL).^22^ This distinction was made to facilitate the training of predictive models specifically geared toward classifying potent AMPs. Based on the specified criteria, the data sets CD90_E_25922, CD90_P_27853, and CD90_S_25923 contain 244, 182, and 228 AMPs in the positive set, respectively. To ensure statistical validity, an equal number of negative AMPs are included in each data set to match the size of their corresponding positive set.

For the hemolysis model, our data set construction methods bear similarities to the approaches adopted by Reymond et al.,^35^ albeit with distinct criteria in place. The positive set (nonhemolytic) was determined based on the minimum hemolytic concentration (MHC) for hemolytic activity against human erythrocytes, which was set at greater than or equal to 128 μg/mL, while the corresponding hemolysis level was required to be less than or equal to 10%. For the negative data set (hemolytic), we set the MHC values against human erythrocytes to be less than or equal to 64 μg/mL, with a hemolysis level set at 50%. Based on the aforementioned criteria, the data set CD90_HE consists of 207 AMPs in the positive set and 207 AMPs in the negative set. The sequences of the CD90 data sets can be found in the Supporting Information (SI).

### Testing Data Set Construction for Evaluation

Two categories of peptide test sets, consisting of peptides with a length of 10–30 amino acids, were utilized to assess the predictive capability of our models. The first category, denoted as CD40, was derived from our collected data sets. These test sets were intentionally excluded from our training set, and the sequence identity between the CD40 test set and the training set was less than 40%. The CD40 test set encompassed four subtest sets, each comprising 10 peptides in the positive set and 10 peptides in the negative set. These subtest sets were designated as follows: (i) CD40_E_25922, based on *E. coli* ATCC 25922 data; (ii) CD40_P_27853, based on *P. aeruginosa* ATCC 27853 data; (iii) CD40_S_25923, based on *S. aureus* ATCC 25923 data; and (iv) CD40_HE, based on human erythrocyte data. The sequences of the CD40 data sets can be found in the SI.

The second category, referred to as NewAMP (see Table S3), comprised AMPs newly published between the years 2022 and 2023, with amino acid lengths shorter than 30. These AMPs were entirely absent from our training set, thus serving as a blind test. The inclusiveness of each NewAMP subset enables us to assess the wide-ranging predictive capability without confining it to a particular strain. The NewAMP test set comprised four subtest sets as well: (i) NewAMP_E, based on *E. coli* data, consisting of 6 peptides in the positive set and 20 peptides in the negative set; (ii) NewAMP_P, based on *P. aeruginosa* data, comprising 3 peptides in the positive set and 12 peptides in the negative set; (iii) NewAMP_S, based on *S. aureus* data, including 8 peptides in the positive set and 22 peptides in the negative set; the positive AMPs have MIC < 10 μg/mL and the negative AMPs have MIC > 100 μg/mL, and (iv) NewAMP_HE, based on human erythrocyte data, with 9 peptides in the positive set (nonhemolysis, 10% hemolysis with MHC > 128 μg/mL) and 2 peptides in the negative set (hemolysis, 50% hemolysis with MHC < 64 μg/mL).

### Features

Prior studies have investigated various properties, such as amphipathicity, charge distribution, helicity, hydrophobicity, and lipophilicity,^16,17^ as well as repeating motifs like arginine–tryptophan^18^ and tryptophan–leucine–lysine,^19^ to enhance the antimicrobial activity of AMPs. However, the application of these properties in innovative peptide design has been shown to be limited.^42^ Since the mechanism of action of AMPs on bacterial membranes is multifaceted, it cannot be solely explained by a single or a few specific properties. Notably, the 3D structure of AMPs provides a more accurate depiction of certain physicochemical properties compared to the 1D amino acid sequence. In this study, we employ two primary categories of features: those based on the 1D amino acid sequence and those based on the 3D structure, to train our predictive models by ML approaches. The 1D sequence-based feature can be further classified into three general types: amino acid composition (AAC), physical–chemical properties (PCP) of the entire peptide, and grouped amino acid distribution (gD). On the other hand, the features based on the 3D structures of AMPs fall into two general types: specific interactions within the peptides themselves and the interactions between AMPs and the bacterial membrane. The list of features utilized in this study and their corresponding abbreviations are presented in Table S1. The features are described as follows.

#### AAC

The amino acid composition (AAC) of AMPs was determined in this study, considering the presence of favorable amino acids within the AMP sequences. In addition to the conventional repertoire of 20 natural amino acids, we accounted for variations at the N-terminal and C-terminal positions, which are frequently subjected to modifications in AMPs. Any variation observed was represented by the value 1, while the absence of variation was denoted by 0. Consequently, the AAC features encompassed a total of 22 variables.

#### PCP

A total of 11 physical–chemical properties (PCPs) were calculated in this study. These include hydrophobicity, charge density, isoelectric point (pI), *in vivo* aggregation, electronic charge index, Boman index, and five *Z* scales. The hydrophobicity values in our study were calculated using the scale provided by Moon et al.,^43^ which is specifically measured at a pH of 3.5. Considering that the hydrophobic sub-moment (HSM) feature (see below) serves to depict the hydrophilicity of peptides in a neutral condition, we integrated the hydrophobicity scale developed by Moon et al.^43^ to capture the chemical space of AMPs within acidic environments for our ML-based search.

Charge density was determined by normalizing the net charge of the AMPs with respect to their sequence length. It has been observed that higher isoelectric points are directly associated with enhanced bactericidal effects, particularly in α-helical AMPs.^44^*In vivo* aggregation was calculated using the method developed by Conchillo-Solé et al., which utilizes an aggregation-propensity scale derived from *in vivo* experiments involving natural amino acids.^45^ The electronic charge index was computed based on the approach devised by Collantes et al.^46^ This method involves summing the absolute values of the CNDO/2 charges of the side chain atoms from the electronic charge scale of natural amino acids. The Boman index serves as a quantitative measure of the binding potential of peptides or proteins to membranes or other receptors.^47^ It is obtained by summing the solubility values of individual amino acids and normalizing the sum by the total number of amino acids. The *Z* scales consist of five distinct scales, denoted as *Z*_1_–*Z*_5_. *Z*_1_ represents hydrophilicity, *Z*_2_ corresponds to steric properties (steric bulk/polarizability), *Z*_3_ represents electronic properties (polarity/charge), and *Z*_4_ and *Z*_5_ represent electronegativity, heat of formation, electrophilicity, and hardness. These five scales were initially generated by Sandberg et al.^48^

#### gD

The grouped amino acid distribution (gD) is a feature employed to assess the arrangement of amino acid patterns within the primary sequence of a protein, taking into account both physicochemical and structural properties. The term “D” denotes the distribution of amino acids possessing a specific property at distinct positions along the sequence, namely, the initial position, 25% position, 50% position (median), 75% position, and the final position.^49^ In this study, we considered six properties: hydrophobicity, normalized van der Waals volume, charge, solvent accessibility, polarizability, and intrapeptide-specific interaction. The properties and their corresponding values are detailed in Table S2.

#### Intrapeptide-Specific Interactions

AMPs typically exhibit amphipathic properties and consist of hydrophobic residues such as Trp, Phe, Leu, Ile, and Val, along with positively charged residues Arg and Lys. Previous research has highlighted the potential of incorporating repeating motifs, such as Arg–Trp^18^ and Trp–Leu–Lys^19^ repeats, in peptide design. In this study, we introduce several intrapeptide-specific interactions as features in our ML approaches. These interactions include cation–π, cation–Leu, Ile, Val, and π–π interactions. The cation in these interactions refers to the cationic residues Arg and Lys, while the π-residues denote Trp, Tyr, and Phe. We calculate the occurrences of cation–π pairs within an amino acid sequence distance of 1–7, accounting for two helical turns. Similarly, we calculate the occurrences of cation–Leu, Ile, Val pairs within an amino acid sequence distance of 1–7, considering two helical turns. Additionally, we count the occurrences of π–π pairs within an amino acid sequence distance of 1–4, accounting for one helical turn. Given that our AMPs adopt a helical structure, the aforementioned calculations are expected to exhibit greater consistency and reliability.

Furthermore, we have incorporated the maximum common subgraphs (MCS) based on 3D helical AMP structures into our ML approaches. The MCS method previously developed and employed by Chandra, Chakravortty, and co-workers^50^ in AMP design allows for the capture of 3D structural characteristics beyond the sequence of AMPs. In this method, the 3D structures of helical AMPs are represented graphically, with individual residues serving as nodes. Each of the 20 natural amino acids is considered as a distinct type of node in the graph representation. Additionally, interresidue interactions are represented as edges, where covalent bonds and backbone hydrogen bonds between residues are modeled as directed edges.

For this study, we utilized a reference data set of helical AMPs with antimicrobial activity (MIC ≤ 10 μg/mL), whose structures were determined using AF2^36^ or by experiments. Each reference data set consists of 91, 41, 45, and 213 AMPs targeting *E. coli* ACTC25922, *P. aeruginosa* ATCC 27853, *S. aureus* ATCC 25923, and human erythrocytes, respectively. These peptides were carefully selected to ensure their absence from the training set and test sets. The predicted AMP structures were evaluated by comparing them with a reference data set using maximum common subgraph matching. The score of the predicted AMP was determined based on the total number of matching nodes between the predicted structure and the reference data set. The identified common subgraphs represent motifs associated with AMP activity, particularly those containing 4, 5, or 6 identical amino acids compared to the reference data set. Subsequently, we implemented a scoring system to assess the matching sets, and the cumulative scores resulted in the generation of three distinct sets of MCS features. The significance of incorporating MCS features lies in their ability to capture common 3D structural motifs, which play a crucial role in determining the antimicrobial activity of peptides against bacterial strains.

#### AMP–Membrane Interactions

Based on the understanding of the AMP mechanism of action on membranes, we conducted calculations related to the transmembrane depth, angle, and transfer energy (Δ*G*_transf_) of AMPs in terms of their 3D structures using the PPM 3.0 method developed by Lomize et al.^51^ In addition, the hydrophobic sub-moment (HSM) is also calculated. The hydrophobic moment is a crucial factor in peptide–membrane interactions, determining the alignment and distribution of hydrophobic residues within a peptide.^52^ It influences the peptide’s ability to interact with lipid membranes, affecting its orientation, stability, and membrane-binding affinity. The HSM represents an optimized version of the mean hydrophobic moment, aiming to capture the distribution of hydrophobic properties within peptide structures.^53^ This concept has demonstrated its effectiveness in the design of membrane-active peptides with desired mode of action.^53^ Unlike the original mean hydrophobic moment, which relies on an idealized two-dimensional (2D) α-helical wheel representation, the HSM incorporates a 3D peptide structure. It is computed by summing the hydrophobic vectors originating from the geometric center of the peptide. This approach accounts for local deviations from the overall amphiphilic distribution of amino acids within the peptide. The HSM is determined by calculating multiple “hydrophobic sub-moments” for overlapping segments of the peptide, employing a sliding window technique. Specifically, HSMs are computed for all overlapping triplets of amino acid residues. We utilized the Eisenberg’s scale for the calculations related to the hydrophobic surface moment (HSM).^54^

The PPM 3.0 method^51^ is utilized to determine the configurations of peptides within curved or planar, single or multiple membranes by optimizing their Δ*G*_transf_ from an aqueous environment to the membrane media. The Δ*G*_transf_ is computed by considering various contributions, including short-range accessible surface area-dependent interactions (such as H-bonds, van der Waals forces, and hydrophobic interactions with the solvent), as well as long-range electrostatic interactions involving dipole moments, charged groups, and the ionization penalty for ionizable groups. Notably, this method accounts for different electrostatic environments, enabling the consideration of both Gram-positive and Gram-negative bacterial membranes, as well as human red blood cells. Gram-positive bacteria and human red blood cells possess a single bilayer membrane, whereas Gram-negative bacteria have an inner and outer membrane forming a double-layered structure. For Gram-negative bacteria, specifically *E. coli* ATCC 25922 and *P. aeruginosa* ATCC 27853, three features based on the 3D structure (transmembrane depth, angle, and transfer energy Δ*G*_transf_) were generated for the outer membrane, and the corresponding three features were generated for the inner membrane. On the other hand, for *S. aureus* ATCC 25923 and human erythrocytes, three features based on the 3D structure (transmembrane depth, angle, and transfer energy Δ*G*_transf_) were generated.

### Algorithm Description

In this study, five ML algorithms were used, including LGBM, SVM, ANN, CNN, and RNN. Cross-validation was employed to validate the models, where 10-fold cross-validation generated 10 different models. Finally, the predicted scores of the 10 models were averaged using bagging to obtain the final predicted score. These five ML algorithms were described as below.

#### Light Gradient Boosting Machine (LGBM)

Light gradient boosting machine (LGBM) is a high-performance gradient boosting framework^55^ developed by Microsoft^56^ for ML tasks with large-scale data and high-dimensional features. It employs histogram-based algorithms, feature optimization, gradient descent, and a leaf-wise growth strategy to improve training and prediction efficiency while maintaining accuracy.^55^

LGBM incorporates histogram optimization, discretizing features to reduce computational complexity while preserving distribution information for enhanced predictive performance. Feature optimization dynamically selects the most relevant features, improving the model’s accuracy. Gradient descent optimizes the training process, minimizing the loss function to reduce prediction errors. The leaf-wise growth strategy generates leaf nodes dynamically, reducing tree depth and improving training and prediction speed.

Compared to traditional methods, LGBM’s leaf-wise strategy achieves faster convergence and better results by greedily selecting features and thresholds that minimize the objective function. It excels in fitting high-dimensional sparse data and handling imbalanced data sets. However, its effectiveness depends on the data set’s distribution. With scalability, customization options, and support for various tasks, LGBM performs well in classification, regression, ranking, and recommendation systems. Its practical value in big data contexts makes it a powerful tool for ML applications.

#### Support Vector Machine

The support vector machine (SVM) is a supervised machine learning algorithm utilized for classification by discovering an optimal hyperplane that separates different classes. In the case of linearly separable data sets, SVM aims to maximize the distance between projected data points of two classes on the hyperplane. However, for data sets that are not linearly separable, SVM incorporates techniques such as soft margin and kernel functions. The fundamental steps of SVM involve: (1) constructing the model by identifying an optimal hyperplane based on a training data set;^56^ (2) defining the hyperplane, which is an *N* – 1 dimensional linear space for linearly separable data sets and incorporates kernel functions and soft margin for linearly nonseparable data sets; (3) determining the optimal hyperplane by maximizing the projected distance between data points for linearly separable data sets, and utilizing soft margin and kernel functions for linearly nonseparable data sets; and (4) predicting the class of new data based on its position relative to the hyperplane.

The SVM algorithm exhibits notable advantages, including its ability to handle high-dimensional data sets and effectively address linearly nonseparable problems through the utilization of kernel functions and soft margins. Furthermore, SVM enhances classification accuracy by selecting optimal hyperparameters using techniques such as cross-validation.

#### Artificial Neutral Network

The ANN is a computational model that emulates the structure and functionality of the neural network in the human brain. ANNs comprise multiple interconnected neurons (nodes) that receive input from other neurons, optimize the output using weights and biases, and transmit the processed information to subsequent layers of neurons. They can be trained using various learning approaches, including supervised learning, unsupervised learning, and reinforcement learning, and find applications in diverse domains such as image recognition, speech recognition, and natural language processing. In the present study, the supervised learning approach is employed to classify the positive and negative data sets.

The fundamental principle of ANNs is rooted in the transmission of information and the adjustment of weights between neurons. Typically, ANNs consist of an input layer, several hidden layers, and an output layer. The input layer receives external information (referred to as features in this study), transforms it into a format suitable for neural processing, and passes it to the subsequent hidden layers. The hidden layers process and transform the information through multiple layers, enabling the fine-tuning of the final output. Finally, the output layer generates the processed information as the ultimate result. Backpropagation, a commonly employed training algorithm, facilitates the training of ANNs. During the training process, ANNs undergo iterative rounds of backpropagation, adjusting the weights and biases based on the discrepancy between the predicted and actual outcomes to enhance the prediction accuracy.^57^ In this study, three hidden layers were employed, with the respective node numbers for the input, three hidden, and output layers being 148 (151 nodes were used for the Gram-negative bacterial models), 128, 128, 256, and 1.

#### Convolutional Neutral Network

CNNs are widely used in image and speech recognition. They excel at feature extraction through convolution operations using small matrices called convolution kernels. These kernels detect various features, preserving spatial relationships. The resulting feature maps undergo nonlinear transformations and are further processed through pooling operations to downsize them. A fully connected layer then classifies the extracted features.^58^ CNNs exhibit a sequential workflow of convolution, activation, and pooling steps, allowing for automatic feature extraction and classification.

In CNNs, convolutional layers consist of multiple kernels generating feature maps, which are fed into subsequent layers. Each kernel is associated with a bias term for optimization. Convolution operations involve weighted sums and activation functions. Pooling operations reduce feature map size, enhancing computational efficiency and combating overfitting. The final step employs a fully connected layer for classification.^59^ In this study, four hidden layers were employed, with the respective node numbers for the input, four hidden, and output layers being 148 (151 nodes were used for the Gram-negative bacterial models), 128, 128, 16, 10, and 1.

#### Recurrent Neural Network

The recurrent neural network (RNN) is a specialized neural network algorithm designed for processing sequential data. Unlike traditional neural networks, RNNs incorporate a cyclic feedback mechanism that allows the network to retain memory of previous inputs, influencing subsequent inputs. The fundamental architecture of an RNN introduces temporal dependencies among neurons while employing a shared set of weights. Sequential data is provided as input to the RNN, where each time step’s input is transformed into a hidden state vector and passed on to the next time step. This hidden state vector serves the dual purpose of storing features specific to the sequential data and serving as the network’s output.^60^

Training RNNs involve using the backpropagation through time (BPTT) algorithm, similar to conventional neural networks. However, the temporal dependencies present in RNNs significantly increase the computational cost of BPTT, making it susceptible to challenges such as gradient vanishing and exploding. To overcome these issues, several improved approaches have been proposed, including long short-term memory (LSTM) and gated recurrent unit (GRU). Additionally, incorporating multiple layers or utilizing bidirectional RNNs can further enhance the model’s performance.^61^ In the current study, a five-layered RNN model was employed, with the respective node numbers for the input, five hidden, and output layers being 148 (151 nodes were used for the Gram-negative bacterial models), 64, 128, 64, 32, 10, and 1.

### Model Explanation: SHAP Value^37^

The increasing complexity of machine learning (ML) models, such as gradient boosting decision trees (GBDT) and Xtreme Gradient Boosting (XGBoost), presents challenges in terms of interpretability, often rendering these models as black boxes. However, the introduction of Shapley Additive exPlanations (SHAP)^37^ has addressed this issue by providing a framework to explain various ML models, thereby enhancing their interpretability.^62^ Originally derived from cooperative game theory, SHAP serves as a comprehensive tool for explanation. For a given data instance represented as *x*_*i*_, where *x*_*i*,*j*_ denotes the *j*th feature of the *i*th data, f(*x*_*i*_) represents the model’s predicted value for the *i*th data, and *f*_base_ represents the base value of the model (i.e., the mean of the dependent variable for all data), the SHAP value can be computed using the following equation: *f*(*x*_*i*_) = *f*_base_ + ∑_*j*=1_^*n*^φ(*x*_*i*,*j*_).

In this equation, φ(*x*_*i*,*j*_) represents the contribution of the *j*th feature in the *i*th data instance to the predicted value. A positive value of φ(*x*_*i*,*j*_) indicates that the *j*th feature enhances the predictive value of the *i*th data instance, while a negative value indicates a reduction in the predictive value. The notable advantage of SHAP is its ability to capture the influence of each feature on individual data instances and quantify both positive and negative contributions. SHAP offers two types of explanations: global explanations, which involve ranking the importance of all features and visually representing their contributions as they vary, and local feature and sample explanations. These explanations enable the understanding of feature influences on predicted values, including single feature or two-feature interactions.

### Performance Evaluation

In this study, the performance of the model is evaluated through the confusion matrix (TP, TN, FP, and FN), where true positive (TP) refers to the number of samples correctly predicted as positive, true negative (TN) refers to the number of samples correctly predicted as negative, false positive (FP) refers to the number of samples incorrectly predicted as positive, and false negative (FN) refers to the number of samples incorrectly predicted as negative. The following are the evaluation indicators.

Accuracy (ACC): it refers to the proportion of all samples correctly classified and can be expressed using the following formula
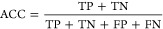
1Precision (PRE): it refers to the proportion of samples correctly predicted as positive out of all samples predicted as positive and can be expressed using the following formula
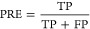
2

Recall (REC): it refers to the proportion of samples correctly predicted as positive out of all actual positive samples and can be expressed using the following formula
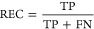
3Specificity (SPE): it refers to the proportion of samples correctly predicted as negative out of all actual negative samples and can be expressed using the following formula
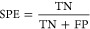
4The Matthews correlation coefficient (MCC) is a performance measure widely used for evaluating binary classification models. Unlike traditional accuracy metrics, MCC incorporates information from true positives, false negatives, false positives, and true negatives in the confusion matrix to produce a single value within the range of −1 to +1. A perfect classifier is indicated by an MCC value of 1, while a value of 0 suggests a classifier that performs no better than random, and −1 signifies a classifier that is completely opposite to random.
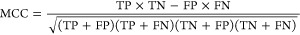
5MCC surpasses accuracy as an evaluation metric, especially for imbalanced class scenarios, as it gives equal weight to both positive and negative classifications. By considering true positives, true negatives, false positives, and false negatives, MCC provides a more comprehensive assessment of model performance. It offers a nuanced evaluation of a binary classifier’s effectiveness, accommodating varying sample sizes across different classes.

The receiver operating characteristic (ROC) curve exhibits the relationship between the true positive rate (TPR) and the false positive rate (FPR). TPR represents the accurate classification of positive samples, while FPR denotes the misclassification of negative samples as positive. AUC, the area under the ROC curve, is a crucial metric ranging from 0.5 to 1.0. A value of 0.5 signifies random classification, whereas 1.0 indicates flawless classification. AUC effectively summarizes the overall performance of a model’s ROC curve into a singular value. Models with ROC curves closer to the upper left corner possess AUC values nearing 1.0, denoting superior performance. Moreover, AUC serves as a comparative measure to assess different models, with higher AUC values suggesting superior performance.

### Determination of MICs of AMPs against *E. coli* and *P. aeruginosa*

*E. coli* DH5a, *P. aeruginosa* ATCC 27853, and PAO1 were used in this study. Briefly, *E. coli* and *P. aeruginosa* strains were first grown in Luria–Bertani (LB) broth at 37 °C for 16 h. Then, overnight bacterial culture was adjusted to OD600 = 0.1, and then diluted 100-fold with LB broth. To determine the MIC of AMPs, 100 μL of different serially diluted AMPs were added into 100 μL of the diluted overnight bacterial culture and further incubated at 37 °C for 16 h. The MIC is defined as the lowest concentration of AMPs that inhibits visible growth of bacteria as observed with the naked eye.

## Results

This study involved the training of 20 predictive models based on 5 ML algorithms, namely, LGBM, SVM, ANN, CNN, and RNN. The purpose of this training was to assess the antimicrobial activity against three specific bacterial strains, namely, *E. coli* ATCC 25922, *P. aeruginosa* ATCC 27853, and *S. aureus* ATCC 25923, as well as the hemolysis of human red blood cells. The bacterial strains encompassed two Gram-negative bacteria, *E. coli* ATCC 25922 and *P. aeruginosa* ATCC 27853, and one Gram-positive bacterium, *S. aureus* ATCC 25923. To evaluate the effectiveness and dependability of the models, we employed the CD40 test set, which possessed a sequence identity 40% lower than that of the training set, as well as the NewAMP test set, consisting of recently published new AMPs.

### Performance of Training Prediction for *E. coli* ATCC 25922

Table 1 provides a detailed overview of the classification performance exhibited by five distinct predictive models, all of which are geared toward *E. coli* ATCC 25922. The training for these models was conducted using the CD90_E_25922 data set, which encompasses a balanced assortment of 244 positive peptides and an equal number of 244 negative peptides, ensuring a comprehensive and unbiased training process. Figure S1 illustrates the evolving loss and receiver operating characteristic (ROC) curves of the ANN model for *E. coli* ATCC 25922 during 10-fold cross-validation. It can be observed that the LGBM model outperforms the other models in all metrics, achieving an accuracy of 80.1%. Particularly, it exhibits higher other metrics compared to those of other models. This suggests that the LGBM model has better discriminative ability for both positive and negative samples, and is particularly effective in detecting positive samples (active antimicrobial peptides). Additionally, the LGBM model obtains higher MCC (0.61) and AUC (0.88) scores than the other models, indicating its superior performance in handling imbalanced data. Furthermore, the ANN and CNN models demonstrate relatively better accuracy (73.1 and 72.9%, respectively) and recall (72.9 and 72.6%, respectively) compared to the RNN model. However, their precision (73.7 and 74.5%, respectively) and specificity (73.6 and 73.9%, respectively) scores are lower than those of the LGBM model. This suggests that these models might be more prone to misclassifying negative samples as positive, but they are effective in detecting true positive samples. Additionally, the MCC and AUC scores of the ANN and CNN models are relatively higher, indicating their ability to distinguish between positive and negative samples. On the other hand, the RNN model performs relatively worse in all metrics. It exhibits lower recall and specificity scores, as well as lower MCC and AUC scores compared to the other models. This suggests that the RNN model has poorer detection capabilities for certain classes and is more prone to misclassifying them as other classes. Therefore, it demonstrates weaker performance in handling imbalanced data.

**Table 1:** Performance of Training Prediction for *E. coli* ATCC 25922

algorithm	accuracy (%)	precision (%)	recall (%)	specificity (%)	MCC	AUC
LGBM	80.1	78.7	81.2	79.5	0.61	0.88
SVM	72.9	75.1	69.3	76.6	0.46	0.73
ANN	73.1	73.7	72.9	73.6	0.46	0.80
CNN	72.9	74.5	72.6	73.9	0.46	0.80
RNN	70.7	73.4	69.6	72.2	0.42	0.75

**Figure 1:**
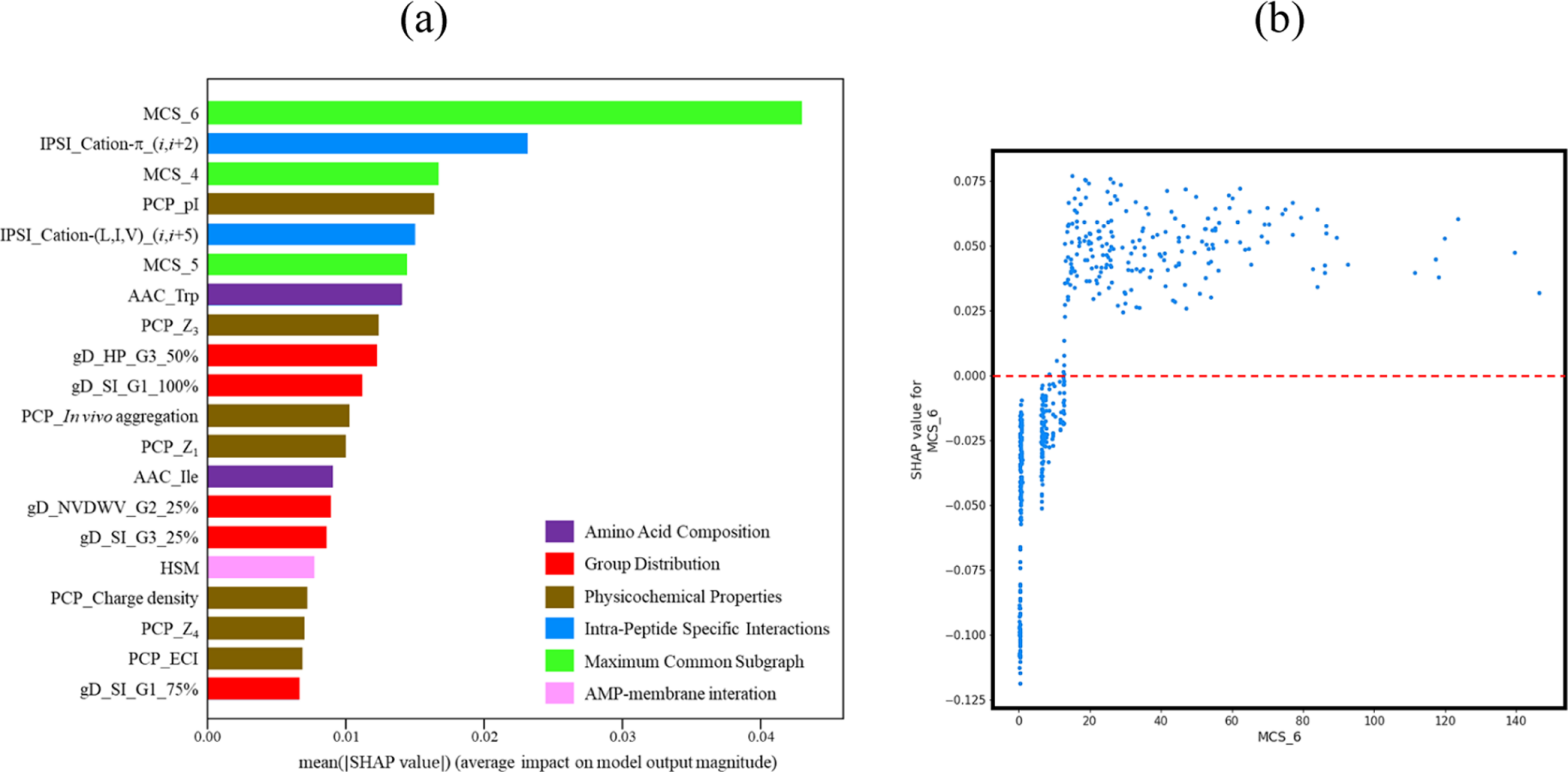
(a) Top 20 feature importance of the LGBM predictive model based on SHAP values for *E. coli* ATCC 25922. Detailed descriptions of the features can be found in Table S1. The color represents the category of the features. (b) Plot of the SHAP values of MCS_6 vs MCS_6 value.

Due to the superior performance of the LGBM predictive model compared to other models, we conducted an analysis of its important features. Figure 1a presents the top 20 feature importance of the optimized LGBM predictive model based on SHAP value with respect to its efficacy against *E. coli* ATCC 25922. Among the identified features, MCS_6, cation–π_(*i*,*i* + 2), and MCS_4 emerge as the three most influential features, all of which exhibit amino-acid-specific interactions. MCS_6 and MCS_4, calculated through the MCS methodology proposed by Nagarajan et al.,^50^ represent the score that indicates the degree to which a peptide shares a subgraph with six and four amino acids, respectively, as compared to a reference data set. The high importance attributed to MCS_6 and MCS_4 suggests that specific structural motifs captured by MCS_6 and MCS_4 play a substantial biological role in determining the antimicrobial activity of peptides against *E. coli*. Moreover, cation–π_(*i*,*i* + 2) signifies the presence of cationic amino acids (e.g., lysine or arginine) and aromatic residues (e.g., Phe, Tyr, or Trp) within the peptide sequence at positions (*i*,*i* + 2). Within a helical structure, the cation–π_(*i*,*i* + 2) motif, which positions two residues on opposing sides of the helix, results in diminished cation–π interactions. This motif underscores the amphiphilic characteristics of the peptide.

Figure 1b illustrates the plot depicting the SHAP values of MCS_6 against the corresponding MCS_6 values. The plot reveals a clear separation of the data into two distinct groups based on an MCS_6 value threshold of approximately 13. It is noteworthy that the majority of data points in the group with MCS_6 values above 13 exhibits positive SHAP values, whereas the majority of data points in the group with MCS_6 values below 13 display negative SHAP values. These findings suggest a meaningful relationship between the presence of common subgroups with the reference set (active antimicrobial peptides) and the antimicrobial activity of a peptide. Peptides that exhibit a higher degree of common subgroups with the reference set are likely to possess enhanced antimicrobial activity. Conversely, peptides with smaller MCS_6 values or a lack of common structural motifs with the reference set are associated with reduced antimicrobial activity.

Additionally, several other factors contribute to the predictive power of the LGBM model. These include physicochemical properties such as the isoelectric point, cation–(L,I,V) (*i*,*i* + 5), and MCS_5. Significantly, all three MCS features play a crucial role in the overall predictive performance of the LGBM model when it comes to determining the antimicrobial activity against *E. coli* ATCC 25922.

### Performance of Training Prediction for *P. aeruginosa* ATCC 27853

Table 2 presents the comparative classification efficacy of five predictive models, each tailored for *P. aeruginosa* ATCC 27853 and trained utilizing the CD90_P_27853 data set. This data set comprises a balanced set of 182 positive and 182 negative peptides, ensuring a robust training environment for the models. Figure S2 presents the evolving loss and ROC curves of the ANN model for *P. aeruginosa* ATCC 27853 throughout 10-fold cross-validation. It is observed that the LGBM model exhibited the highest accuracy, reaching an impressive 82.1%. The SVM model followed closely, while the ANN and CNN models performed comparably, and the RNN model demonstrated the lowest accuracy. Regarding precision and recall, the LGBM model achieved the highest performance with scores of 82.4 and 82.3%, respectively, indicating its superior ability to discriminate positive samples. The other models exhibited relatively poorer performance, with the ANN and CNN models displaying similar results, the SVM model slightly outperforming the ANN and CNN models, and the RNN model performing the worst. The performance of specificity mirrored that of precision, with the LGBM model achieving the highest score of 83.4%, indicating its capability to distinguish negative samples effectively and minimize false positives. The SVM model followed closely, while the ANN and CNN models exhibited comparable performance, and the RNN model showed the poorest specificity.

**Table 2:** Performance of Training Prediction for *P. aeruginosa* ATCC 27853

algorithm	accuracy (%)	precision (%)	recall (%)	specificity (%)	MCC	AUC
LGBM	82.1	82.4	82.3	83.4	0.65	0.95
SVM	78.6	79.3	78.0	79.1	0.58	0.79
ANN	76.6	77.0	76.8	78.0	0.54	0.85
CNN	77.2	76.4	78.3	77.0	0.55	0.84
RNN	71.4	66.0	76.0	69.7	0.44	0.78

From the MCC perspective, the LGBM model demonstrated the best performance with an MCC score of 0.65, signifying its superior handling of imbalanced data. In terms of AUC, the LGBM model exhibited the highest performance with an AUC score of 0.95, followed by the ANN and CNN models, while the SVM and RNN models showed relatively poorer performance. Considering all of the aforementioned metrics, it can be concluded that the LGBM model outperformed the other models, demonstrating superior performance. The SVM model, ANN model, and CNN model exhibited relatively similar performance, while the RNN model performed the worst.

**Figure 2:**
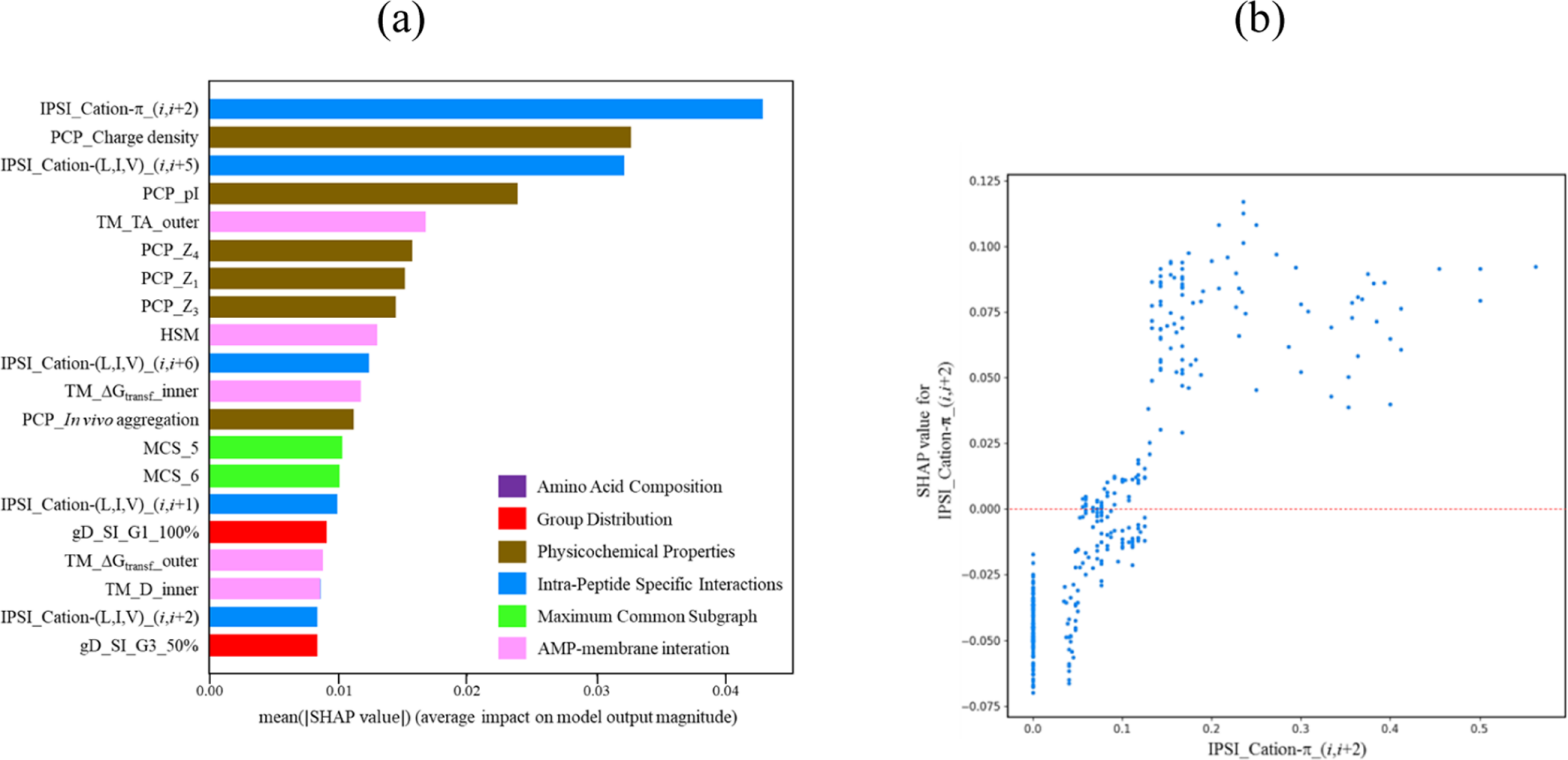
(a) Top 20 feature importance of the LGBM predictive model based on SHAP values for *P. aeruginosa* ATCC 27853. Detailed descriptions of these features can be found in Table S1. The color represents the category of the features. (b) Plot of the SHAP values of cation–π_(*i*,*i* + 2) versus the cation–π_(*i*,*i* + 2) value.

Figure 2a showcases the top 20 feature importance of the optimized LGBM predictive model based on SHAP values pertaining to its effectiveness against *P. aeruginosa* ATCC 27853. The analysis highlights the significance of intrapeptide-specific AA pairs, such as cation–π_(*i*,*i* + 2) and cation–(L,I,V)_(*i*,*i* + 5) as well as physicochemical properties like isoelectric point and charge density, in influencing the antimicrobial activity of peptides against *P. aeruginosa* ATCC 27853. Specifically, the cation–π_(*i*,*i* + 2) feature represents a pairing of cationic residues at position “*i*” and aromatic residues (*i*+2, Phe, Trp, and Tyr), and the cation–(L,I,V) (*i*,*i* + 5) feature represents a combination of cationic residues (i.e., Arg and Lys) at position *i* and hydrophobic residues (*i*+5, Leu, Ile, and Val). The presence of such amino acid pairs contributes to the amphiphilicity of helical peptides, wherein these motifs point the cationic and aromatic/hydrophobic residues on different sides of the helix. The features cation–π_(*i*,*i* + 2) and cation–(L,I,V)_(*i*,*i* + 5) also demonstrate significant importance in the LGBM predictive model against *E. coli* ATCC 25922. The prominence of the isoelectric point and charge density highlights the prevalence of cationic characteristics in the majority of AMPs.

Figure 2b illustrates the plot of the SHAP values of cation–π_(*i*,*i* + 2) versus the cation–π (*i*,*i* + 2) value. The data reveals that samples lacking or exhibiting low cation–π_(*i*,*i* + 2) pairs are associated with negative SHAP values, whereas samples featuring high cation–π_(*i*,*i* + 2) pairs display positive SHAP values. These findings emphasize the significant contribution of cation–π (*i*,*i* + 2) pairs to the antimicrobial activity.

### Performance of Training Prediction for *S. aureus* ATCC 25923

Table 3 displays performance metrics of five models for *S. aureus* ATCC 25923, trained on CD90_S_25923 with 228 positive and negative peptides each. Figure S3 presents the evolving loss and ROC curves of the ANN model for *S. aureus* ATCC 25923 throughout 10-fold cross-validation. Accuracy was a common metric among all models, with LGBM demonstrating the best performance at 78.9%. ANN and CNN exhibited similar performance, achieving accuracies of 71.2 and 70%, respectively. In contrast, SVM and RNN had lower performances at 69.9 and 66.4%, respectively. LGBM performed the best with precision and recall scores of 80.7 and 78.2%, respectively. LGBM achieved the highest specificity at 80.3%. SVM had slightly lower specificity at 67.9%. ANN and CNN showed similar performance in specificity, with CNN performing slightly better. In terms of MCC, LGBM performed the best at 0.58. LGBM demonstrated the best performance in terms of AUC at 0.90. Overall, LGBM exhibited the best performance across these metrics, while RNN performed the worst. Among these models, ANN and CNN showed relatively similar performance, while SVM had slightly lower performance across multiple metrics.

**Table 3:** Performance of Training Prediction for *S. aureus* ATCC 25923

algorithm	accuracy (%)	precision (%)	recall (%)	specificity (%)	MCC	AUC
LGBM	78.9	80.7	78.2	80.3	0.58	0.90
SVM	69.9	69.5	71.9	67.9	0.40	0.70
ANN	71.2	71.9	71.0	71.8	0.43	0.78
CNN	70.0	70.6	69.8	70.9	0.40	0.75
RNN	66.4	70.7	65.8	67.8	0.33	0.69

**Figure 3:**
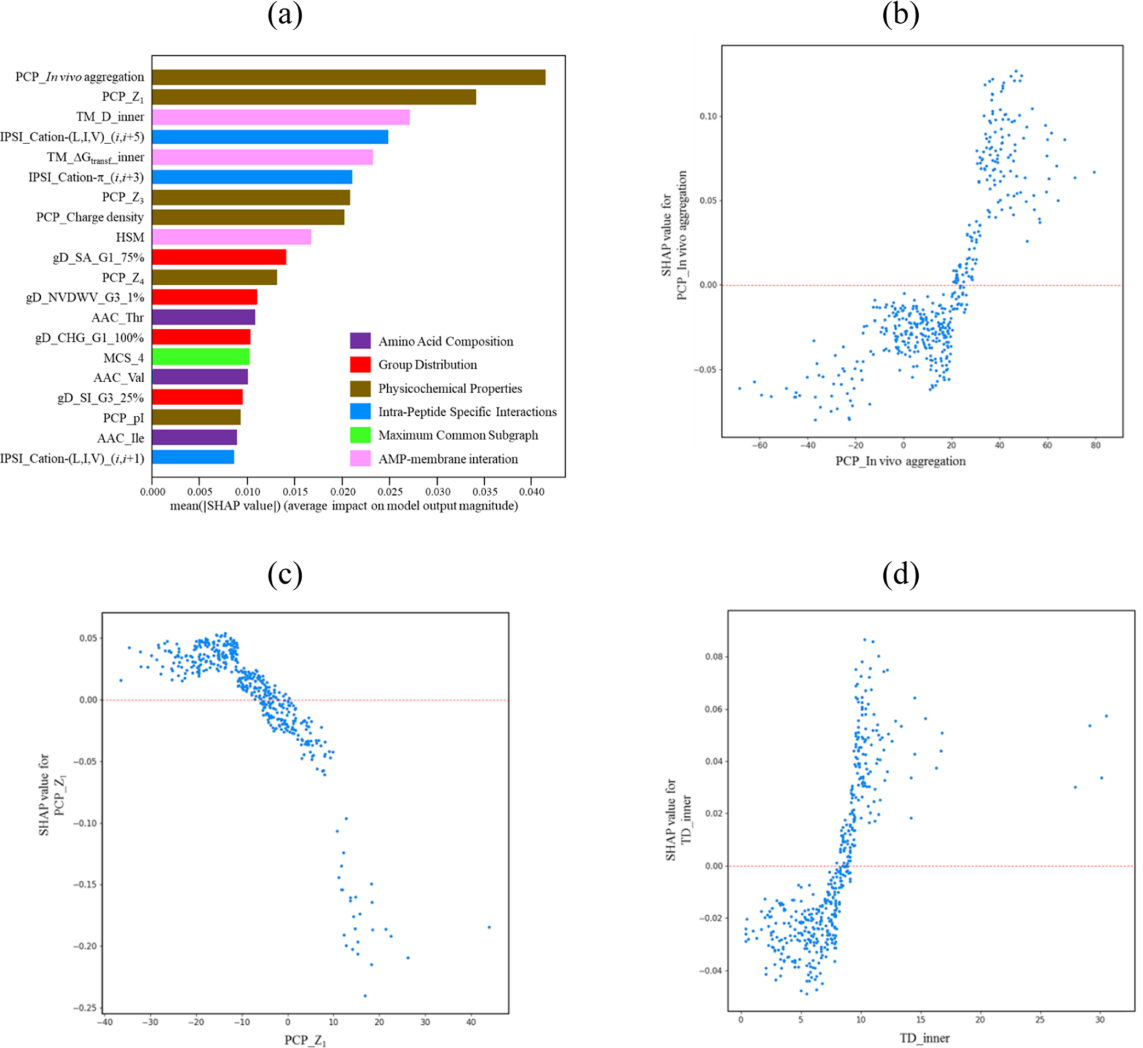
(a) Top 20 feature importance of the LGBM predictive model for *S. aureusATCC 25923*. Detailed descriptions of these features can be found in Table S1. The color represents the category of the features. (b) Plot of the SHAP values of *in vivo* aggregation versus *in vivo* aggregation. (c) Plot of the SHAP values of *Z*_1_ versus *Z*_1_ value and (d) plot of the SHAP values of TM_D_inner versus TM_D_inner value.

Figure 3a presents the top 20 feature importance of the optimized LGBM predictive model, with a focus on the effectiveness against *S. aureus* ATCC 25923. Notably, the analysis highlights the five most significant features associated with the physicochemical properties of the entire peptide. These features include *in vivo* aggregation and *Z*_1_ value, which capture the propensity for peptide aggregation and hydrophobicity, respectively. Additionally, the model assigns importance to transmembrane properties such as membrane insertion depth and transfer free energy, along with the intrapeptide-specific pair, cation–(L,I,V)(*i*,*i* + 5). Furthermore, cation–π(*i*,*i* + 3) pairs are identified as important features contributing to the model’s predictive performance.

Figure 3b provides insights into the distribution of *in vivo* aggregation values within the training set and their relationship with the corresponding SHAP values. The plot reveals the existence of two distinct distributions, each associated with different contributions to the classification of the positive and negative sets. Notably, peptides exhibiting significantly higher *in vivo* aggregation values align with positive SHAP values, while the distribution characterized by smaller *in vivo* aggregation values corresponds to negative SHAP values. Similarly, Figure 3c explores the distribution of *Z*_1_ values within the training set and their association with the corresponding SHAP values. It is observed that samples with negative *Z*_1_ values (indicating greater hydrophobicity) tend to have positive SHAP values, suggesting their propensity to approach the membrane. Conversely, samples with positive *Z*_1_ values (indicating higher hydrophilicity) exhibit negative SHAP values, indicating their inclination to remain in the aqueous phase. Furthermore, Figure 3d delves into the distribution of TM_D_inner values within the training set and their relationship with the corresponding SHAP values. The plot demonstrates that samples with larger TM_D_inner values (reflecting deeper insertion into the membrane) exhibit positive SHAP values. On the other hand, samples with smaller TM_D_inner values (indicating a preference for the membrane surface) display negative SHAP values. Given that the action mechanism of antimicrobial peptides involves targeting the membrane, the transmembrane insertion depth plays a pivotal role in determining their antimicrobial activity.

### Performance of Training Prediction for Hemolysis in Human Erythrocytes

Table 4 details the classification outcomes of five predictive models targeting human erythrocytes, utilizing the CD90_HE data set. Figure S4 presents the evolving loss and ROC curves of the ANN model for human erythrocyte hemolysis throughout 10-fold cross-validation. This data set comprises a balanced selection of 207 AMPs in both the positive and negative sets, ensuring equitable model training. LGBM demonstrated the best performance with an accuracy of 86.0%, MCC of 0.73, AUC of 0.99, and both recall and specificity reaching 86.6%. Among all models, RNN exhibited the poorest performance, achieving only 74.2% accuracy. Its MCC was 0.486, AUC was 0.784, recall was 73.2%, and specificity was 75.9%. This may be attributed to the characteristics of RNN, which result in weaker long-term dependency memory capacity. Overall, LGBM exhibited the best performance, while RNN performed the poorest.

**Table 4:** Performance of Training Prediction for Hemolysis in Human Erythrocytes

algorithm	accuracy (%)	precision (%)	recall (%)	specificity (%)	MCC	AUC
LGBM	86.0	85.6	86.6	86.6	0.73	0.94
SVM	80.2	80.9	80.3	80.2	0.61	0.80
ANN	81.9	81.2	82.9	82.2	0.65	0.90
CNN	78.8	77.9	79.7	78.9	0.58	0.85
RNN	74.2	76.2	73.2	75.9	0.49	0.78

**Figure 4:**
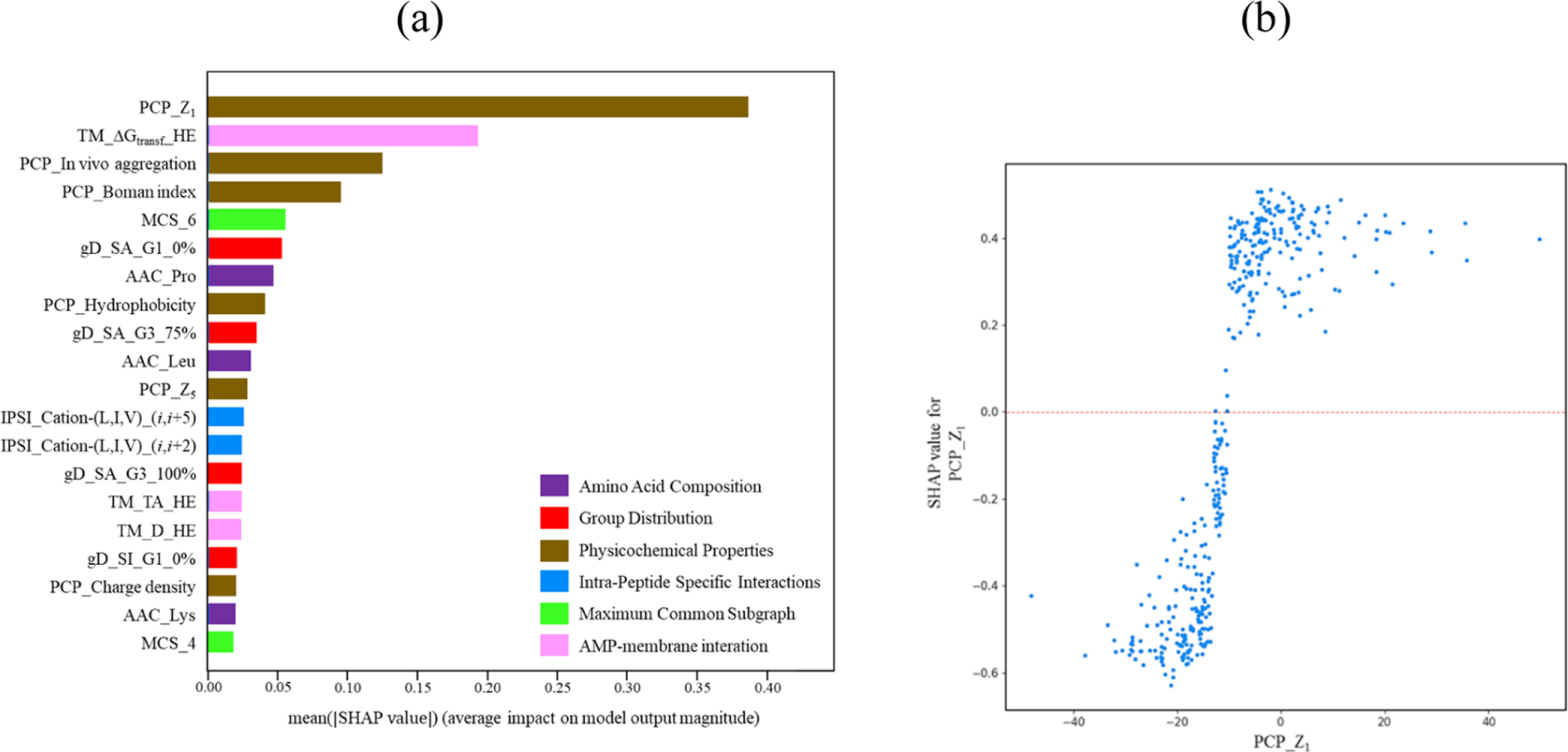
(a) Top 20 feature importance of the LGBM predictive model for human erythrocytes. Detailed descriptions of these features can be found in Table S1. The color represents the category of the features. (b) Plot of the SHAP values of *Z*_1_ vs *Z*_1_ values.

Figure 4a presents the feature importance analysis of the LGBM predictive model based on SHAP values concerning human erythrocytes. The analysis reveals that the *Z*_1_ feature exhibits significantly higher importance compared to other features. These results are distinct different from the feature importance of the three LGBM predictive models against bacterial, in which feature importance is not predominated by a single feature. The *Z*_1_ feature, represented in *Z* scales, signifies the hydrophilicity of the peptide. In addition, the second and third important features are transmembrane free energy and *in vivo* aggregation, respectively.

Figure 4b depicts the distribution of *Z*_1_ values within the training set in relation to SHAP values. The analysis reveals two distinct distributions: one with predominantly positive *Z*_1_ values, corresponding to positive SHAP values, and another with mostly negative *Z*_1_ values, corresponding to negative SHAP values. This finding suggests that peptides with positive *Z*_1_ values play a crucial role in the LGBM model’s classification of the positive class (nonhemolysis). On the other hand, peptides with negative *Z*_1_ values play a crucial role in the LGBM model’s classification of the negative class (hemolysis). Given the substantial dependency of hemolysis prediction on the *Z*_1_ feature, along with the relatively minor influence of other features, it is evident that the LGBM model for predicting hemolysis demonstrates superior performance compared to the three LGBM models designed for bacterial prediction.

### Testing of Predictive Models

The generalization ability of our predictive models was assessed using CD40 test sets in order to determine their effectiveness in screening new and *de novo* APMs that exhibit a sequence space distinct from our training set, which comprises known AMPs. The predictive results for the four CD40 test subsets, obtained from our models, are presented in Table 5. It is evident that the prediction accuracies achieved by the LGBM predictive model are comparable to those observed for their corresponding training sets. However, the SVM predictive model demonstrates a limited ability to predict the CD40_S_25923 set accurately, achieving a prediction accuracy of only 55.0%. Among the three neural network models, the ANN model outperforms the others. Given the relatively low reliance of the CD40 test sets on the training data, our LGBM predictive model exhibits potential for exploring novel AMP activity spaces rather than being limited to the known space.

**Table 5:** Results of Prediction Accuracy (%) on the CD40 Test Sets of AMPs for Each Training Model

target bacterial	test set	LGBM	SVM	ANN	CNN	RNN
*E. coli* ATCC 25922	CD40_E_25922	75.0	75.0	80.0	55.0	75.0
*P. aeruginosa* ATCC 27853	CD40_P_27853	75.0	65.0	70.0	60.0	60.0
*S. aureus* ATCC 25923	CD40_S_25923	75.0	55.0	70.0	70.0	55.0
human erythrocytes	CD40_HE	85.0	80.0	80.0	85.0	75.0

Table 6 presents the predictive outcomes for the four subsets of the NewAMP test (see Table S3), as obtained from our models. It is evident that the LGBM predictive model outperforms the other three models in terms of performance. Specifically, the LGBM predictive model demonstrates notable accuracy in predicting the NewAMP_E, NewAMP_P, and NewAMP_S test sets, achieving accuracies of 92.3, 100, and 83.3%, respectively. These results suggest that the LGBM predictive model, based on a specific bacterial strain, possesses the ability to accurately predict other strains as well. Interestingly, with regard to the NewAMP_HE test set, LGBM and ANN predictive models exhibit identical predictive accuracy of 81.8% and the other three models exhibit identical predictive accuracy of 72.7%.

**Table 6:** Results of Prediction Accuracy (%) on the NewAMP Test Sets of AMPs for Each Training Model

target bacterial	test set	LGBM	SVM	ANN	CNN	RNN
*E. coli*	NewAMP_E	92.3	61.5	76.9	73.1	73.1
*P. aeruginosa*	NewAMP_P	100	73.3	73.3	73.3	86.7
*S. aureus*	NewAMP_S	83.3	76.7	73.3	73.3	80.0
human erythrocytes	NewAMP_HE	81.8	72.7	81.8	72.7	72.7

Furthermore, we conducted a comprehensive evaluation of the state-of-the-art AMP predictors, DBAASP,^10,22^ using the NewAMP test sets, as summarized in Table 7. The DBAASP predictor^10,22^ exhibited satisfactory predictive accuracy across different subsets of NewAMP, namely, NewAMP_E, NewAMP_P, and NewAMP_S, achieving accuracies of 69.2, 86.7, and 76.7%, respectively. These results demonstrate comparable accuracy to our neural network models. Both our LGBM model and the DBAASP model^10,22^ demonstrate strong performance in their respective contexts, underlining their effectiveness in AMP prediction. It is important to note that the accuracy of the DBAASP predictor was calculated based on our classification criteria of active AMPs (MIC < 10 μg/mL) versus nonactive AMPs (MIC > 100 μg/mL). It is worth mentioning that the DBAASP predictor was initially developed using positive peptides with MIC values below 25 μg/mL, while our tests involved positive peptides with even lower MIC values (below 10 μg/mL). However, it is important to note that most AMP predictors, like the AMP Scanner,^24^ AmPEP,^32^ and CAMP,^63^ do not integrate MIC values of peptides into their models; instead, they rely on AMP vs non-AMP classification criteria. Consequently, directly comparing the performance of these models with our model and the DBAASP model can be challenging.

**Table 7:** Results of Prediction Accuracy (%) on the NewAMP Test Sets of AMPs for our LGBM Model and DBAASP^22^ Predictor

target bacterial	test set	our LGBM model	DBAASP^22^
*E. coli*	NewAMP_E	92.3^a^	69.2^a^
*P. aeruginosa*	NewAMP_P	100^a^	86.7^a^
*S. aureus*	NewAMP_S	83.3^a^	76.7^a^

a Accuracy calculated based on our active (MIC < 10 μg/mL) vs nonactive (MIC > 100 μg/mL) MIC criteria.

### AMP Design and *In Vitro* Antimicrobial Activity Test

To showcase an application of our predictive models developed in this study, we *in silico* engineer the PEM-2 antimicrobial peptide to against *E. coli*, *P. aeruginosa* ATCC 27853, and *PA*01 by our LGBM predictive model. PEM-2 is a synthetic 13-AA peptide variant derived from myotoxin II, a homologue of phospholipase A2 found in the venom of the Bothrops asper snake.^64^ Previous studies have demonstrated the potent antimicrobial activity of PEM-2, which is attributed to its amphiphilic properties. Helix wheel of PEM-2 (sequence: KKWRWWLKALAKK) shows it has amphiphilic property (refer to Figure S5). To this end, we engineer the amino acids on the amphiphilic interface. We first single mutate five amino acids (R4, W6, A9, A11, and K13) on the hydrophobic and hydrophilic interface of PEM-2 generating 95 (5 × 19 AA) peptides and submit them to our LGBM predictive model. R4 mutating to R, N, or Q, W6 mutating to L, I, V, R, K, F, and Y, A9 mutating to F, G, H, I, K, L, M, N, Q, R, S, T, V, W, and Y, A11 mutating to F, G, H, I, K, L, M, N, Q, R, S, T, V, W, and Y, and K13 mutating to H, N, Q, and R give higher scores than that of PEM-2 and are saved for further multiple mutations.

Multiple mutations with mutations on the above ones give 40,960 peptides and these peptides are submitted to our LGBM for further prediction. 1319 of these peptides have higher scores than that of PEM-2. Seven peptides out of a pool of 1319 were chosen for further evaluation of their antibacterial effects through the measurement of MIC, as detailed in Table 8. The criteria for choosing these peptides were rigorous, guaranteeing a thorough and meticulous evaluation: (1) they possessed higher scores in our evaluations; (2) they exhibited a significant number of mutation points, with a minimum criterion of at least two; and (3) the mutated amino acids in these peptides showcased distinct physical–chemical properties, setting them apart not only from PEM-2 but also from each other. It is noteworthy that six out of these seven peptides, as indicated in Table 8, feature four mutation points, resulting in a substantial alteration in their amino acid composition.

**Table 8:** Measured MIC Values (μg/mL) of PEM-2 and Seven Designed Peptides at 150 mM NaCl Concentrations

name	sequence	*E. coli* DH5a	*P. aeruginosa* ATCC 27853	*PA*01
PEM-2	KKWRWWLKALAKK	50	>50	>50
PEM-2_325	KKWRWWLKILAKR	50	>50	>50
PEM-2_3435	KKWRWILKRLWKR	25	50	>50
PEM-2_3039	KKWRWILKKLYKQ	12.5^a^	>50	>50
PEM-2_1812	KKWRWFLKLLRKH	25^a^	>50^b^	>50^b^
PEM-2_4982	KKWRWKLKWLIKH	12.5^a^	>50	>50
PEM-2_8505	KKWRWVLKRLIKR	25	>50^b^	>50
PEM-2_2150	KKWRWFLKRLVKR	50	>50^b^	>50

a Partial inhibitory effect at next lower concentration.

b Partial inhibitory effect at this concentration.

For validation purposes, we synthesized eight AMPs including PEM-2 predicted by our models and subjected them to antimicrobial assays against three Gram-negative bacteria: *E. coli* DH5a, *P. aeruginosa* ATCC 27853, and *PA*01. Table 8 presents the assessed MIC values of our designed peptides against *E. coli* DH5a, *P. aeruginosa* ATCC 27853, and *PA*01. It is worth noting that a previous study conducted under different culturing conditions reported lower MIC values for PEM-2 (MIC values approximately 6.25 μg/mL for *E. coli* ATCC 25925 and 12.5 μg/mL for *P. aeruginosa* ATCC 27853)^65^ compared to our findings. In this context, we undertake a comparative analysis and discussion of the antimicrobial activities of both PEM-2 and our designed peptides under the same culturing conditions. In the case of *E. coli* DH5a, PEM-2 showcases an MIC value of 50 μg/mL. Remarkably, our designed peptides demonstrate equivalent or lower MICs compared to PEM-2. Specifically, peptides p12_3039 and p12_4982 display impressive MIC values below 12.5 μg/mL against *E. coli* DH5a. Turning our attention to *P. aeruginosa* ATCC 27853, the measured MIC for PEM-2 was found to exceed 50 μg/mL. Impressively, three of our designed peptides outperformed PEM-2, with peptide p12_3435 exhibiting an MIC value of 50 μg/mL, thereby surpassing the performance of PEM-2. For strain PA01, we measured the MIC of PEM-2 to be greater than 50 μg/mL. Our designed peptide, p12_1812, demonstrated marginally superior performance than PEM-2. Collectively, these results strongly underscore the potential of our predictive models in bolstering the design and enhancement of peptides with AMPs. The ability of our model to design peptides with competitive MIC values holds promise for the development of effective AMPs against high-priority pathogens.

## Discussion

The integration of a task-oriented strategy for the discovery of potential AMPs against drug-resistant strains necessitates the creation of a methodological framework. This should be capable of efficiently scanning the vast space of amino acid sequences, accurately predicting antimicrobial activity, and discerning peptides active against specific strains. Furthermore, it is crucial that this approach yields interpretable results to elucidate the mechanistic action of AMPs.^66,67^ In this study, LGBM and SVM were used as classification algorithms, while ANN, VNN, and RNN were employed as neural network algorithms to predict the antimicrobial activity. Notably, LGBM demonstrated superior performance compared to the other algorithms. The exceptional predictive capabilities of LGBM highlight the effectiveness and efficiency of the LGBM algorithm in antimicrobial activity classification. LGBM’s ability to handle large data sets, address imbalanced data, and capture intricate feature relationships contributes to its success in antimicrobial activity prediction.

The discovery of novel AMPs that combat drug-resistant strains necessitates the establishment of predictive methodologies capable of identifying active peptides outside the known AMP sequence space.^9^ To date, the bulk of ML models are constructed to predict AMP activities leveraging known AMP sequences.^24,26,32^ Given that AMPs operate based on their 3D structures, physicochemical properties derived from these structures present viable data for ML algorithms to learn and extrapolate the antimicrobial space beyond the confines of the known sequence space.^31,66^ The rapid advancements in predicting proteins’ 3D structures from their amino acid sequences, as seen in DeepMind’s AF2^36^ and Baker et al.’s ROSETTA,^68^ provide highly accurate representations of peptide and protein 3D structures. By integrating these 3D structures into ML models, the predictive capacity can be broadened to encompass unknown AMP spaces, potentially accelerating the development of potent AMPs.

The development and evaluation of AMP predictors are crucial for advancing our understanding of AMPs and their potential applications in combating microbial infections. In this context, various AMP predictors have been developed using different data sets, peptide lengths, ML algorithms, and criteria for defining AMPs. However, assessing the performance of these predictors is often limited to test sets, without considering the similarity between the training and test data. Building on this approach, we have utilized an unbiased test set comprising newly published AMPs that were not included in our training set. This approach allows us to evaluate the performance of our predictors in a more rigorous and realistic manner. In this regard, a comparative study can be performed on published web-tools of AMP predictors. Moreover, we have employed the CD40 test set, which exhibits a significantly lower sequence identity compared to the peptides in our training set. This test set provides a valuable opportunity to evaluate the generalizability of our predictors and assess their capability to predict the antimicrobial activities of peptides with distinct sequences. Our predictive models have demonstrated favorable performance in predicting the antimicrobial activities of the CD40 test set, highlighting their ability to effectively explore and predict the activity of de novo AMPs with unique and diverse sequences. The utilization of unbiased test sets and the evaluation of performance on the CD40 test set offer important insights into the generalizability and robustness of our predictors. These findings enhance our confidence in the predictive capabilities of our models and their potential for guiding the design and development of novel antimicrobial peptides.

The significant features identified for different bacterial models can serve as valuable indicators to investigate and comprehend the diverse modes of action of AMPs against various bacterial species. A comparative analysis of the top five significant features, based on SHAP values from Figures 1 to 4, reveals noteworthy insights into our predictive LGBM models for two Gram-negative bacteria, *E. coli* ATCC 25922 and *P. aeruginosa* ATCC 27853, as well as the Gram-positive bacterium *S. aureus* ATCC 25923. Interestingly, common important features such as cation–π_(*i*,*i* + 2), isoelectric point (pI), and cation–(L,I,V)_(*i*,*i* + 5) are observed in the predictive models for Gram-negative bacteria *E. coli* and *P. aeruginosa*, while are distinctly different from Gram-positive bacterium *S. aureus*. Prior investigations have shown that the isoelectric point (pI) distribution of ribosomal AMPs can be adequately approximated by the summation of two Gaussian curves, with peaks at larger pI values. These curves represent one lower peak centered around pI values of 14 and another higher peak centered around pI values of 10^7^. Moreover, a study by Ghazvini and colleagues,^44^ analyzing 22 AMPs with anti-*Helicobacter pylori* effects, revealed that the majority of these AMPs exhibited an α-helical structure and possessed cationic properties characterized by high positive charges and isoelectric points. It is worth noting that *Helicobacter pylori* is a Gram-negative bacterial species. According to the helix wheel model, the cation–π_(*i*,*i* + 2) and cation–(L,I,V)_(*i*,*i* + 5) motifs in α-helical AMPs exhibit a distinctive arrangement. Cationic residues are positioned on one side of the helix, while hydrophobic and aromatic residues, namely, Leu, Val, Ile, Phe, Trp, and Tyr are situated on the other side, leading to an amphiphilic property. The amphipathic nature of these motifs is particularly noteworthy, as it plays a significant role in the antimicrobial activity of α-helical AMPs targeting Gram-negative bacteria. By displaying cationic and hydrophobic moieties on opposing or other side of the helix, these motifs allow the AMPs to effectively interact with the bacterial membrane, disrupting its integrity and leading to antimicrobial action.^69^ This amphipathic configuration is considered crucial for the selective targeting and effective penetration of the bacterial membrane, highlighting its importance in the design and function of AMPs against Gram-negative bacteria.^11^

The five most important features identified by our predictive LGBM models for the Gram-positive bacterium *S. aureus* ATCC 25923 are as follows: *in vivo* aggregation, *Z*_1_, TM_D_inner, cation–(L,I,V)_(*i*,*i* + 5), and TM_Δ*G*^transfer^_inner. Notably, with the exception of cation–(L,I,V)_(*i*,*i* + 5), these features are distinct from those observed in the model for Gram-negative bacteria, namely, *E. coli* ATCC 25922 and *P. aeruginosa* ATCC 27853. This observation suggests potential differences in the underlying action mechanisms of AMPs between Gram-negative and Gram-positive bacteria. The features TM_D_inner and TM_Δ*G*_transfer__inner are particularly relevant to the mode of action of AMPs, as they respectively represent the depth of peptide insertion into the membrane and the transfer free energy of peptides from the aqueous phase to the membrane. These characteristics are directly associated with the mechanism by which AMPs target and interact with the bacterial membrane. Additionally, *in vivo* aggregation reflects the tendency of AMPs to aggregate within the membrane, potentially leading to pore formation.^70^ Moreover, the feature *Z*_1_ indicates the partition tendency of AMPs between the aqueous phase and the membrane.^71^ The differential importance of these features between Gram-negative and Gram-positive bacterial models highlights the complexity and specificity of AMP action against different types of bacteria.^72^ Understanding these distinctions is crucial for tailoring and optimizing AMP design for effective antimicrobial strategies against specific bacterial types.

The prominent features identified by our predictive LGBM model for hemolysis in human erythrocytes demonstrate distinct characteristics compared to the three bacterial models. Notably, the *Z*_1_ values exert a dominant influence over the other features, and the SHAP values of the remaining features exhibit a rapid decrease. These findings are explicable considering the fundamental differences between the zwitterionic nature of the human erythrocyte membrane and the negatively charged membranes of bacteria. The analysis reveals two discernible distributions (Figure 4b): one characterized by predominantly positive *Z*_1_ values, corresponding to positive SHAP values, and another characterized by mostly negative *Z*_1_ values, corresponding to negative SHAP values. This observation implies that peptides with positive *Z*_1_ values play a pivotal role in the LGBM model’s classification of the positive class (nonhemolysis) and *vice versa*. As such, the *Z*_1_ values of AMPs serve as crucial determinants of their hemolytic activity against human erythrocytes.^73^ Moreover, the simplicity of the *Z*_1_ values of peptides renders them valuable chemical rules for the rapid design of nonhemolytic AMPs with enhanced safety profiles for potential therapeutic applications.

## Conclusions

We have developed advanced predictive ML models in terms of 3D helical structure-based features targeting WHO priority pathogens. These models prioritize shorter AMPs, focus on specific bacterial strains, use a positive set with low MIC values, and evaluate their impact on human erythrocytes. Our rigorous validation, employing the LGBM learner, confirms an accuracy exceeding 75%. Independent testing, utilizing one test set with less than 40% sequence identity to the training set and one test set including newly published AMPs, enhances the credibility of our models. Our models assist in designing new AMPs derived from PEM-2, with some demonstrating superior effectiveness compared to the parent peptide. SHAP analysis highlights key features, revealing differences in AMP action mechanisms between Gram-negative and Gram-positive bacteria. Our approach offers practical applications while acknowledging model limitations, including applicability only to α-helices, reliance on encoded amino acids, and pH variations, which we are actively addressing.

## Data Availability

To streamline the utilization of our predictive models, we have established a web server named AMP-META (AMP Explorer and Toxicity Analyzer). This platform enables the convenient submission and prediction of AMPs based on their PDB structures. Access to these web-based prediction tools (20 unique models) can be found at http://ai-meta.ncu.edu.tw/amp-meta/. The sequences and activities for the CD90, CD40, and NewAMP data sets in CSV format and their 3D structures in PDB format are available from https:/github.com/LCCBTsai/AMP_ML.

## Supporting Information Available

List of all of the features used in this study (Table S1); distribution features encompass various groups of physicochemical properties (Table S2); list of the sequence and activity of the NewAMP database (Table S3); evolving loss and ROC curves of the ANN model for *E. coli* ATCC 25922, *P. aeruginosa* ATCC 27853, *S. aureus* ATCC 25923, and human erythrocyte hemolysis, respectively (Figures S1–S4), and helix wheel of PEM-2 (Figure S5) (PDF)
