# Supplementary material for: Accelerating Antimicrobial Peptide Discovery for WHO Priority Pathogens through Predictive and Interpretable Machine Learning Models
Source: ACS Omega. 2024 Feb 13;9(8):9357–74. doi: 10.1021/acsomega.3c08676 (PMC10905719; doi:10.1021/acsomega.3c08676)
Supplement: Supplementary file 1 — ao3c08676_si_001.pdf [file ao3c08676_si_001.pdf]

Supporting Information

Accelerating Antimicrobial Peptide Discovery for WHO Priority Pathogens through Predictive and Interpretable Machine Learning Models

Cheng-Ting Tsai, Chia-Wei Lin, Gen-Lin Ye, Shao-Chi Wu, Philip Yao,

Ching-Ting Lin*, Lei Wan* and Hui-Hsu Gavin Tsai*

Correspondence:

Prof. Hui-Hsu Gavin Tsai: Email: hhtsai@cc.ncu.edu.tw

Prof. Ching-Ting Lin orcid: Email: gingting@mail.cmu.edu.tw

Prof. Lei Wan: Email: leiwan@cmu.edu.tw

Table S1 A comprehensive list of features used in this study along with their corresponding abbreviations.

**Feature Type & Abbreviation**	**#**	**Abbreviation**	**Description**
Amino Acid Composition (AAC)	22	AAC_AA 3 letter code<br>AAC_Ncap<br>AAC_Ccap	AAC_Ncap indicates N-terminal is modified<br>AAC_Ccap indicates C-terminal is modified
Group Distribution (gD)	90	gD_property_group number_*n*%	For the details of property and group number, please see Table S2.<br>0%=1st AA<br>100%=last AA
Physicochemical Properties (PCP)	11	PCP_property	Property:<br>Hydrophobicity<br>Charge density<br>Isoelectric point (pI)<br>In vivo aggregation<br>Electronic charge index (ECI)<br>Boman index<br>Z_1: hydrophilicity<br>Z_2: steric properties (steric bulk/polarizability)<br>Z_3: electronic properties (polarity/charge)<br>Z_4 and Z_5: electronegativity, heat of formation, electrophilicity, and hardness

Intra-Peptide Specific Interactions (IPSI)	18	IPSI_Specific Interaction_(*i*,*i*+*n*)	Specific Interaction:<br>π−π pairs<br>Cation-π pairs<br>Cation-(L,I,V) pairs
Maximum Common Subgraph (MCS)	3	MCS_*n*	*n* = 4,5,6<br>shares a subgraph with *n* amino acids.
Transmembrane depth (TM_D)	2(1)	TM_D _X	X=Inner:<br>Gram-negative bacteria inner membrane<br>and<br>Gram-positive bacteria inner membrane<br><br>X=Outer:<br>Gram-negative bacteria outer membrane<br><br>X=HE<br>Human erythrocytes membrane
Transmembrane tilt angle (TM_TA)	2(1)	TM_TA _X	
Transmembrane (TM) energy	2(1)	TM_ΔG_{transf}_X	
Hydrophobic Submoment (HSM)	1	HSM	

Table S2 The distribution features encompass various groups of physicochemical properties.

Physicochemical property	***Group 1 (G1)***	***Group 2 (G2)***	***Group 3 (G3)***
***Hydrophobicity* (HP)**	**Polar** (D, E, K, N, Q, and R)	**Neutral** (A, G, H, P, S, T, and Y)	**Hydrophobic** (C, F, I, L, M, V, and W)
***Normalized VDW Volume* (NVDWV)**	**0-2.78** (A, C, D, G, P, S, and T)	**2.95-4.0** (E, I, L, N, Q, and V)	**4.03-8.08** (F, H, K, M, R, W, and Y)
Charge (CHG)	**Positively** (K, R, and H)	**Neutral** (A, C, F, G, I, L, M, N, P, Q, S, T, V, W, and Y)	**Negatively** (D and E)
Solvent accessibility (SA)	**Buried** (A, C, F, G, I, L, V, and W)	**Exposed** (D, E, K, N, R, and Q)	**Intermediate** (H, M, P, S, T, and Y)
Polarizability (Pol)	**0–0.108** (G, A, S, D, and T)	**0.128–0.186** (C, P, N, V, E, Q, I, and L)	**0.219–0.409** (K, M, H, F, R, Y, and W)
**Specific Interaction (SI)**	**π-ring/ring** (Y, F, W, and H)	**H-Bond/Charge** (S, T, D, E, K, N, Q, and R)	**Alkyl Chain** (P, G, A, C, I, L, M, and V)

Table S3 NewAMP database[1-9]

Sub-set	Name	Sequence	Bacterial Strain	MIC (μg/mL)
NewAMP_E	HJH-5[8]	KKLLRLLKVLLR	*E. coli* ATCC 25922	1.5
NewAMP_E	RaCa-2[3]	FFPIIARLAAKVIPSLVCAVTKKC	*E. coli* ATCC 25922	4-16
NewAMP_E	RaCa-6[3]	ATAWRIPPPGMQPIIPIRIRPLCGKQ	*E. coli* ATCC 25922	No inhibition
NewAMP_E	RaCa-8[3]	FPAIICKVSKNC	*E. coli* ATCC 25922	No inhibition
NewAMP_E	RaCa-10[3]	ALVAKIQKFPVFNTLKLCKLELEII	*E. coli* ATCC 25922	No inhibition
NewAMP_E	Hydr_2[5]	GVAKKLLWIAAKKPAGAGSKFKLL-NH_2	*E. coli* ATCC 25922	512
NewAMP_E	Hydr_3[5]	GELKKLWQAGKLSEEDGGAFKAG-NH_2	*E. coli* ATCC 25922	512
NewAMP_E	Hydr_4[5]	FLPLIGRVFSGIL-NH_2	*E. coli* ATCC 25922	512
NewAMP_E	Hydr_5[5]	FLPLIGRVFSGIK-NH_2	*E. coli* ATCC 25922	512
NewAMP_E	Hydr_6[5]	FLPLIGRVLSGIA-NH_2	*E. coli* ATCC 25922	512
NewAMP_E	Hydr_7[5]	FLPLIGRVKSGIK-NH_2	*E. coli* ATCC 25922	512
NewAMP_E	Hydr_8[5]	FLPIKNRYASAAE-NH_2	*E. coli* ATCC 25922	512
NewAMP_E	KS22[4]	KLKKVTGKKMSKCMKCKIYVCS	*E. coli*	633.82
NewAMP_E	RD10[4]	RTLFVCRVGD	*E. coli*	291.34
NewAMP_E	FE23[4]	FTFYLPLFVCRRNPRPRRVSCRE	*E. coli*	728.36
NewAMP_E	Hm-AMP[2]	RLKRFKRVALRREKTARNFRSIVS	*E. coli* MG 1655	298.86
NewAMP_E	Hm-AMP2[2]	EKRWRRLIFNYF	*E. coli* MG 1655	7.95
NewAMP_E	Hm-AMP3[2]	VVKTGCQRRRMLPR	*E. coli* MG 1655	170.01
NewAMP_E	Hm-AMP5[2]	FIFSKLFLGLIKI	*E. coli* MG 1655	153.90
NewAMP_E	Hm-AMP7[2]	GLKITHTITVKGILGFLWVKIVAQK	*E. coli* MG 1655	276.44
NewAMP_E	Hm-AMP9[1]	WGKNLQMKSLYNNLTIGHYKRRF	*E. coli* MG 1655	286.84
NewAMP_E	hHK-1[8]	TGKASQFFGLM-NH_2	*E. coli* ATCC 25922	151.63
NewAMP_E	AH-1[8]	LKKWTGKASQFFGLM-NH_2	*E. coli* ATCC 25922	111.35
NewAMP_E	AH-3[8]	LKKWLKKWTGKASQFFGLM-NH_2	*E. coli* ATCC 25922	4.59
NewAMP_E	AH-4[8]	LKKWLKKWTLKASQFFGLM-	*E. coli* ATCC 25922	4.70

		NH_2		
NewAMP_E	AH-5[8]	LKKWLKKWTPKASQFFGLM-NH_2	*E. coli* ATCC 25922	4.67
NewAMP_P	RaCa-2[3]	FFPIIARLAAKVIPSLVCAVTKKC	*P. aeruginosa* ATCC 10148	128
NewAMP_P	RaCa-4[3]	FLTFPGMTFGKLLGK	*P. aeruginosa* ATCC 10148	No inhibition
NewAMP_P	RaCa-6[3]	ATAWRIPPPGMQPIIPIRIRPLCGKQ	*P. aeruginosa* ATCC 10148	No inhibition
NewAMP_P	RaCa-7[3]	FFPRVLPLANKFLPTIYCALPKSVGN	*P. aeruginosa* ATCC 10148	No inhibition
NewAMP_P	RaCa-8[3]	FPAIICKVSKNC	*P. aeruginosa* ATCC 10148	No inhibition
NewAMP_P	RaCa-9[3]	FYFPVSRKFGGK	*P. aeruginosa* ATCC 10148	No inhibition
NewAMP_P	RaCa-10[3]	ALVAKIQKFPVFNTLKLCKLELEII	*P. aeruginosa* ATCC 10148	No inhibition
NewAMP_P	RaCa-11[3]	SNRDFFKVNIFRLCG	*P. aeruginosa* ATCC 10148	No inhibition
NewAMP_P	PEP-38[1]	GLKDWVKKALGSLWKLANSQKAIISGKKS	*P. aeruginosa*	No inhibition
NewAMP_P	hHK-1[8]	TGKASQFFGLM-NH_2	*P. aeruginosa* ATCC 27853	151.63
NewAMP_P	AH-1[8]	LKKWTGKASQFFGLM-NH_2	*P. aeruginosa* ATCC 27853	222.71
NewAMP_P	AH-2[8]	LKKWTLKASQFFGLM-NH_2	*P. aeruginosa* ATCC 27853	114.94
NewAMP_P	AH-3[8]	LKKWLKKWTGKASQFFGLM-NH_2	*P. aeruginosa* ATCC 27853	9.18
NewAMP_P	AH-4[8]	LKKWLKKWTLKASQFFGLM-NH_2	*P. aeruginosa* ATCC 27853	4.70
NewAMP_P	AH-5[8]	LKKWLKKWTPKASQFFGLM-NH_2	*P. aeruginosa* ATCC 27853	4.67
NewAMP_S	HJH-3[8]	KLLKRKLLVTLR	*S. aureus* ATCC 29213	6.25
NewAMP_S	HJH-4[8]	KLLKRKLLVLLR	*S. aureus* ATCC 29213	3
NewAMP_S	HJH-5[8]	KKLLRLLKVLLR	*S. aureus* ATCC	1.5

			29213	
NewAMP_S	HJH-6[8]	KKLLKKLLRLLKVLLR	*S. aureus* ATCC 29213	0.75
NewAMP_S	GW18[6]	GWGAKRWGKRGWKWKRHW-$COONH_2$	*S. aureus* ATCC 6538	3.12
NewAMP_S	RaCa-2[3]	FFPIIARLAAKVIPSLVCAVTKKC	*S. aureus* ATCC 6538P	2-4
NewAMP_S	RaCa-4[3]	FLTFPGMTFGKLLGK	*S. aureus* ATCC 6538P	No inhibition
NewAMP_S	RaCa-6[3]	ATAWRIPPPGMQPIIPIRIRPLCGKQ	*S. aureus* ATCC 6538P	No inhibition
NewAMP_S	RaCa-7[3]	FFPRVLPLANKFLPTIYCALPKSVGN	*S. aureus* ATCC 6538P	No inhibition
NewAMP_S	RaCa-8[3]	FPAIICKVSKNC	*S. aureus* ATCC 6538P	No inhibition
NewAMP_S	RaCa-9[3]	FYFPVSRKFGGK	*S. aureus* ATCC 6538P	No inhibition
NewAMP_S	RaCa-10[3]	ALVAKIQKFPVFNTLKLCKLELEII	*S. aureus* ATCC 6538P	No inhibition
NewAMP_S	RaCa-11[3]	SNRDFFKVNIFRLCG	*S. aureus* ATCC 6538P	No inhibition
NewAMP_S	WSKK11[9]	WSKKWKKKWKW-NH_2	*S. aureus* TISTR 746	8
NewAMP_S	KS22[4]	KLKKVTGKKMSKCMKCKIYVCS	*S. aureus*	633.82
NewAMP_S	RD10[4]	RTLFVCRVGD	*S. aureus*	291.34
NewAMP_S	FE23[4]	FTFYLPLFVCRRNPRPRRVSCRE	*S. aureus*	728.36
NewAMP_S	Hm-AMP1[2]	RLKRFKRVALRREKTARNFRSIVS	*S. aureus* ST 88	128.51
NewAMP_S	Hm-AMP3[2]	VVKTGCQRRRMLPR	*S. aureus* ST 88	170.01
NewAMP_S	Hm-AMP5[2]	FIFSKLFLGLIKI	*S. aureus* ST 88	153.90
NewAMP_S	Hm-AMP6[2]	WITLKRLGRCHPWGGHGH	*S. aureus* ST 88	211.15
NewAMP_S	Hm-AMP7[2]	GLKITHTITVKGILGFLWVKIVAQK	*S. aureus* ST 88	276.44
NewAMP_S	Hm-AMP8[2]	RAVIYKIPYNAIASRWIIAPKKC	*S. aureus* ST 88	267.53
NewAMP_S	Hm-AMP9[2]	WGKNLQMKSLYNNLTIGHYKRRF	*S. aureus* ST 88	286.84

NewAMP_S	Hm-AMP10[2]	VGALAGFLYWHFLRKGTKMVGK	*S. aureus* ST 88	248.00
NewAMP_S	hHK-1[8]	TGKASQFFGLM-NH_2	S. aureus ATCC 25923	151.63
NewAMP_S	AH-1[8]	LKKWTGKASQFFGLM-NH_2	S. aureus ATCC 25923	222.71
NewAMP_S	AH-2[8]	LKKWTLKASQFFGLM-NH_2	S. aureus ATCC 25923	229.89
NewAMP_S	AH-3[8]	LKKWLKKWTGKASQFFGLM-NH_2	S. aureus ATCC 25923	9.18
NewAMP_S	AH-4[8]	LKKWLKKWTLKASQFFGLM-NH_2	S. aureus ATCC 25923	4.70
**Sub-set**	**Name**	**Sequence**	**Erythrocyte**	**MHC(μg/mL) (% hemolysis)**
NewAMP_HE	HJH-1[8]	KLLKHKLLVTLA	*Erythrocyte*	382.72[a]
NewAMP_HE	HJH-2[8]	KLLKHKLLVTLR	*Erythrocyte*	382.72[a]
NewAMP_HE	HJH-3[8]	KLLKRKLLVTLR	*Erythrocyte*	382.72[a]
NewAMP_HE	HJH-4[8]	KLLKRKLLVLLR	*Erythrocyte*	382.72[a]
NewAMP_HE	HJH-6[8]	KKLLKKLLRLLKVLLR	*Erythrocyte*	3.95 (90%)
NewAMP_HE	Hm-AMP4[2]	FILYGLIRFGRLLRK	*Erythrocyte*	18.65 (77%)
NewAMP_HE	Hm-AMP8[2]	RAVIYKIPYNAIASRWIIAPKKC	*Erythrocyte*	535.06 (6.50%)
NewAMP_HE	hHK-1[8]	TGKASQFFGLM-NH_2	*Erythrocyte*	473.84 (5.05%)
NewAMP_HE	AH-1[8]	LKKWTGKASQFFGLM-NH_2	*Erythrocyte*	695.96 (1.01%)
NewAMP_HE	AH-3[8]	LKKWLKKWTGKASQFFGLM-NH_2	*Erythrocyte*	918.12 (7.88%)
NewAMP_HE	AH-5[8]	LKKWLKKWTPKASQFFGLM-NH_2	*Erythrocyte*	934.12 (2.94%)

a. % Hemolysis data was not reported in the original paper[8]; it was considered as non-hemolysis. Therefore, we have also classified them as non-hemolysis.

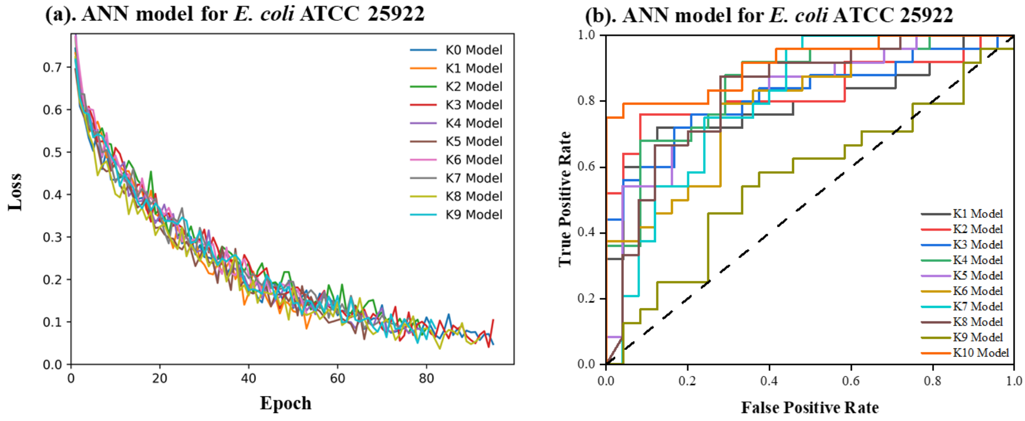

Figure S1 The evolving (a). loss and (b). ROC curves of the ANN model for *E. coli* ATCC 25922 during 10-fold cross-validation. It is observed that the evolution of loss values indicates a steady decrease and stabilization, suggesting an effective learning process throughout the training. ROC curves approach the top-left corner, signify a high classification performance of the model.

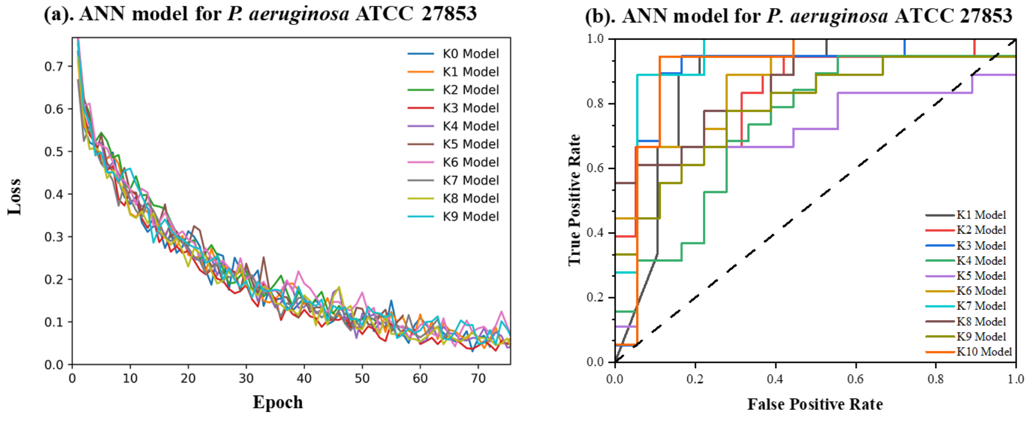

Figure S2 The evolving (a). loss and (b). ROC curves of the ANN model for *P. aeruginosa* ATCC 27853 during 10-fold cross-validation. The decreasing and stabilizing loss values signify effective learning during training, and ROC curves approaching the top-left corner indicate high classification performance.

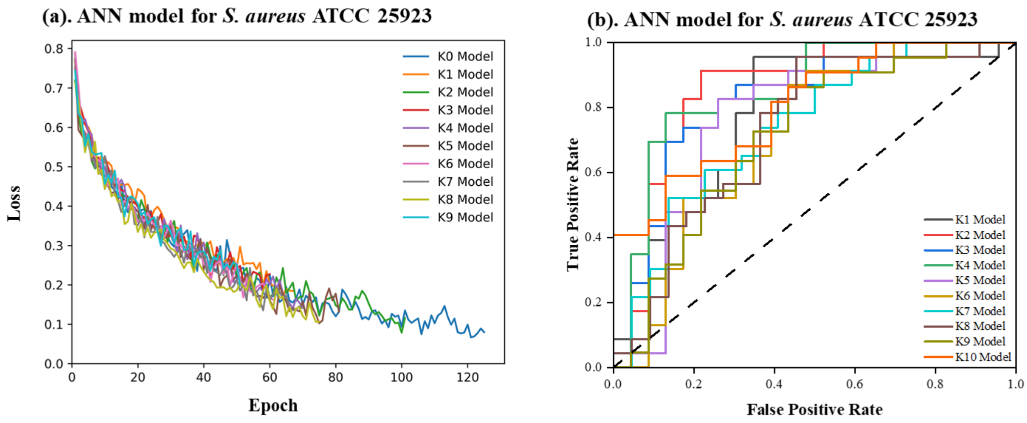

Figure S3 The evolving (a). loss and (b). ROC curves of the ANN model for *S. aureus ATCC 25923* during 10-fold cross-validation. The decreasing and stabilizing loss values indicate an effective learning process during training, and ROC curves approaching the top-left corner signify high classification performance.

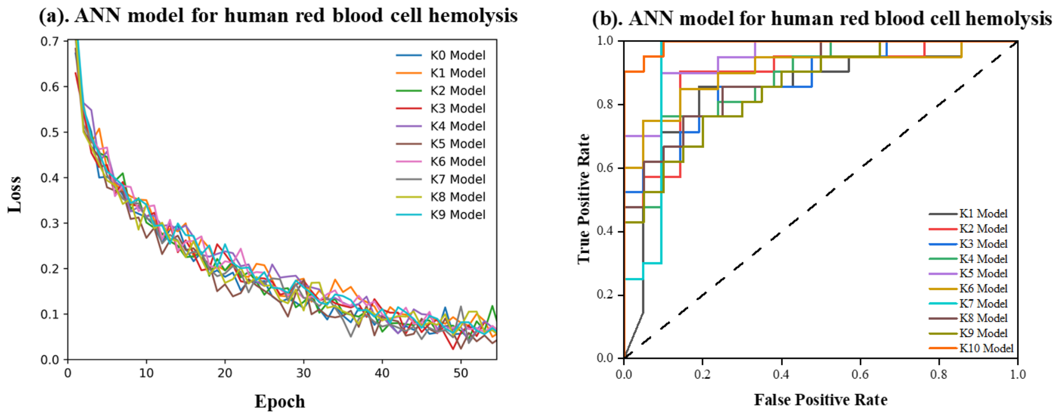

Figure S4 The evolving (a). loss and (b). ROC curves of the ANN model for human red blood cell hemolysis during 10-fold cross-validation. The decreasing and stabilizing loss values indicate an effective learning process during training, and ROC curves approaching the top-left corner signify high classification performance.

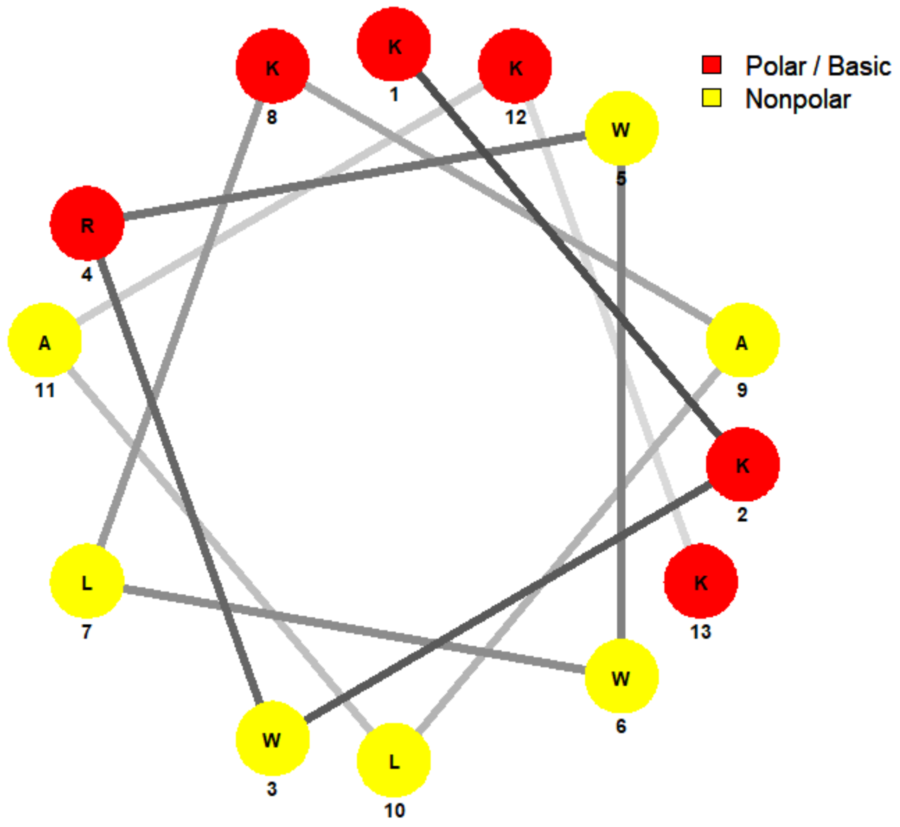

Figure S5 Helix wheel of PEM-2 generated by NetWheels tool[10].

References:

1. Bolatchiev, A.; Baturin, V.; Shchetinin, E.; Bolatchieva, E., Novel Antimicrobial Peptides Designed Using a Recurrent Neural Network Reduce Mortality in Experimental Sepsis. *Antibiotics (Basel)* **2022,** *11* (3).
2. Grafskaia, E. N.; Pavlova, E. R.; Latsis, I. A.; Malakhova, M. V.; Ivchenkov, D. V.; Bashkirov, P. V.; Kot, E. F.; Mineev, K. S.; Arseniev, A. S.; Klinov, D. V.; Lazarev, V. N., Non-toxic antimicrobial peptide Hm-AMP2 from leech metagenome proteins identified by the gradient-boosting approach. *Materials & Design* **2022,** *224*, 111364.
3. Li, C.; Sutherland, D.; Hammond, S. A.; Yang, C.; Taho, F.; Bergman, L.; Houston, S.; Warren, R. L.; Wong, T.; Hoang, L. M. N.; Cameron, C. E.; Helbing, C. C.; Birol, I., AMPlify: attentive deep learning model for discovery of novel antimicrobial peptides effective against WHO priority pathogens. *BMC Genomics* **2022,** *23* (1), 77.
4. Ruiz Puentes, P.; Henao, M. C.; Cifuentes, J.; Muñoz-Camargo, C.; Reyes, L. H.; Cruz, J. C.; Arbeláez, P., Rational Discovery of Antimicrobial Peptides by Means of Artificial Intelligence. *Membranes (Basel)* **2022,** *12* (7).
5. Szymczak, P.; Możejko, M.; Grzegorzek, T.; Bauer, M.; Neubauer, D.; Michalski, M.; Sroka, J.; Setny, P.; Kamysz, W.; Szczurek, E., HydrAMP: a deep generative

model for antimicrobial peptide discovery. *bioRxiv* **2022**, 2022.01.27.478054.
6. Yuan, B.; Lu, X.; Yang, M.; He, Q.; Cha, Z.; Fang, Y.; Yang, Y.; Xu, L.; Yan, J.; Lai, R.; Wang, A.; Yu, X.; Duan, Z., A designed antimicrobial peptide with potential ability against methicillin resistant Staphylococcus aureus. *Frontiers in Microbiology* **2022,** *13*.
7. Wang, Y.; Zhu, G.; Wang, W.; Zhang, Y.; Zhu, Y.; Wang, J.; Geng, M.; Lu, H.; Chen, Y.; Zhou, M.; Chen, J.; Zhang, F.; Yang, J.; Cheng, X., Rational design of HJH antimicrobial peptides to improve antimicrobial activity. *Bioorganic & Medicinal Chemistry Letters* **2023,** *83*, 129176.
8. Yao, Y.; Zhang, W.; Li, S.; Xie, H.; Zhang, Z.; Jia, B.; Huang, S.; Li, W.; Ma, L.; Gao, Y.; Song, J.; Wang, R., Development of Neuropeptide Hemokinin-1 Analogues with Antimicrobial and Wound-Healing Activity. *Journal of Medicinal Chemistry* **2023,** *66* (10), 6617-6630.
9. Theansungnoen, T.; Phosri, S.; Bumrungthai, S.; Daduang, J.; Klaynongsruang, S.; Daduang, S., Novel non-cytotoxic antimicrobial peptides WSKK11 and WSRR11 with potent activity against Cutibacterium acnes. *J Antimicrob Chemother* **2022,** *77* (4), 1012-1019.
10. Mól, A.; S. Castro, M.; Fontes, W., *NetWheels: A web application to create high quality peptide helical wheel and net projections*. 2018.
